# Elucidating the role of genetic background in shaping the flavor characteristics of Panxian dry-cured ham: A multidimensional approach integrating sensory evaluation, GC-IMS, and GC–MS

**DOI:** 10.1016/j.fochx.2026.104595

**Published:** 2026-10-08

**Authors:** Dong Li, Ying Huang, Jun Lv, Chi Zhao, Chunlin Zhang, Hong Zhang, Yu Mu

**Affiliations:** a School of Food Engineering, Moutai Institute, Renhuai 564507, China; b Guizhou Engineering Research Center for Health-Functional Liquor Brewing Technology, Moutai Institute, Renhuai 564507, China; c School of Brewing Engineering, Moutai Institute, Renhuai 564507, China

**Keywords:** Panxian ham, Pig genotype, Sensory evaluation, Characteristic aroma compounds, Multivariate statistical analysis

## Abstract

This study characterized flavor differences among Panxian dry-cured hams produced from purebred Pingdi pigs (PDZ), Pingdi × Landrace (CBZ), and Pingdi × Duroc (DLK). Quantitative descriptive analysis confirmed significant breed-driven disparities in fatty, mushroom, fruity, and aged aromas despite shared fundamental ham flavor traits. In total, 77 and 58 volatiles were detected by gas chromatography–ion mobility spectrometry and gas chromatography–mass spectrometry, respectively, with merely nine overlapping compounds, from which multivariate statistical analysis prioritized 24 and 17 candidate discriminatory volatiles. Aroma activity indices and partial least squares regression illustrated potential aroma-active compounds for each group: lipid oxidation-derived aldehydes/alcohols may dominate CBZ's fatty and mushroom aromas; unique esters may contribute to PDZ's floral-fruity aroma; and accumulated ketones may be associated with DLK's intense aged aroma. This work demonstrates that pig genetic background is an important regulator of ham volatile profiles, contributing to quality control in Panxian ham production.

## Highlights

• PDZ showed fruity aroma, CBZ prominent fatty aroma, and DLK presented aged aroma.
• Dual GC-IMS and GC–MS platforms mapped flavor profiles of Panxian dry-cured hams.
• LEfSe and PLS-DA identified 24 and 17 candidate discriminatory volatiles, respectively.
• PLSR established the relationship between genetic background and sensory differences.

## Graphical abstract

**Figure:**
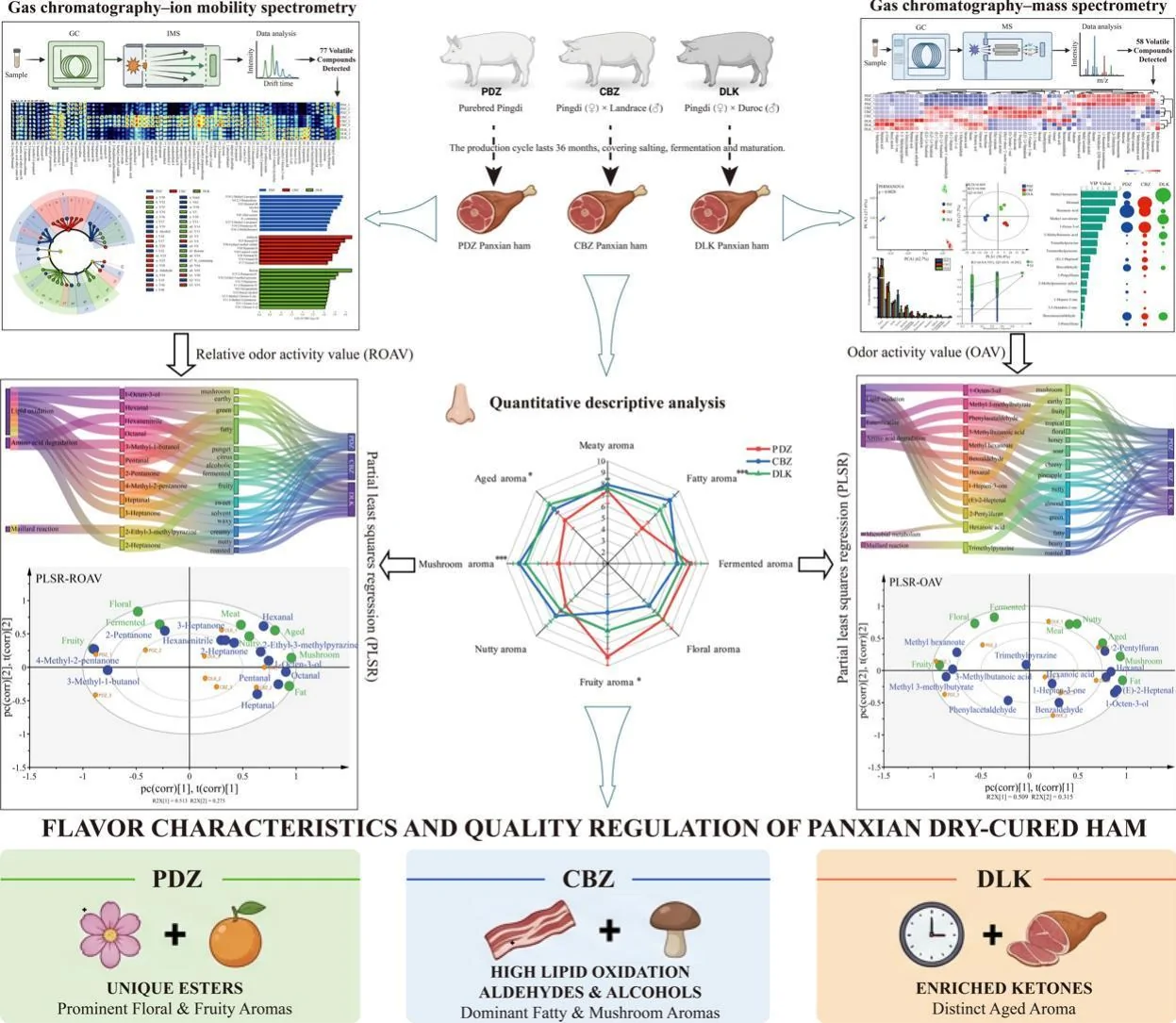

## 1 Introduction

Dry-cured ham is a widely consumed traditional fermented meat product, and its distinctive flavor serves as a core indicator for quality evaluation and commercial value. The formation of ham flavor is a complex dynamic biochemical process that relies on the synergy of multiple pathways, including lipid oxidation, protein degradation, Maillard reaction, and microbial metabolism. Meanwhile, it is regulated by various factors such as pig breeds, processing technologies, and environmental conditions (Li et al., 2023). Previous studies have shown that genetic differences among pig breeds directly alter fundamental indicators of muscle tissue, including lipid composition, endogenous enzyme activity, and flavor precursor contents (Regmi et al., 2025), which further lead to differences in the sensory quality of ham products (Savić et al., 2025).

Panxian ham is the third dry-cured ham in China to be recognized as a nationally protected geographical indication product (Mu et al., 2020). The designated local breed used for its production is the Pingdi pig (also known as Kele pig), a member of the Wujin pig group. The Pingdi pig is characterized by high intramuscular fat content and superior meat quality, which provide an important basis for the characteristic flavor of Panxian ham. To balance growth performance and product quality, crossbreeding between Pingdi pigs and introduced breeds such as Landrace and Duroc has been gradually promoted in local breeding and processing industries. Although the effects of pig breed or genetic background on the volatile profiles of dry-cured hams have been reported for several traditional ham products, most studies focused on a single production system or relied predominantly on one analytical platform (Savić et al., 2025; Wen et al., 2024). In addition, previous studies have generally focused either on volatile composition or on sensory properties, whereas the direct relationship between genetic background, sensory phenotype, volatile markers, and aroma activity has not been systematically established for Panxian ham (Mu et al., 2026; Zheng et al., 2020). Therefore, systematic comparisons between purebred Pingdi pigs and their crossbred progeny under the identical Panxian ham production process are of great importance, as they are key to industrial-scale expansion and standardized production.

A single analytical technique cannot fully characterize the overall flavor profile of complex food matrices. Gas chromatography–ion mobility spectrometry (GC-IMS) requires minimal sample pretreatment and possesses high detection sensitivity. It can establish flavor fingerprints and is suitable for rapid screening and dynamic monitoring of flavor differences (Guo et al., 2025). Gas chromatography–mass spectrometry (GC–MS) features superior separation performance and a comprehensive standard spectral library and is recognized as a classic method for analyzing complex volatile components (Quintanilla-Casas et al., 2025). The combined application of the two techniques can integrate their advantages, greatly expand the detection range and improve the identification accuracy of volatile flavor compounds (Cai et al., 2024). Additionally, threshold-based evaluation via odor activity value (OAV) and relative odor activity value (ROAV) facilitates the screening of characteristic aroma compounds, and these two indices have been widely adopted in the flavor evaluation of various premium hams (Liu et al., 2009; Xiao et al., 2024).

Therefore, in this study, hind legs from Pingdi pigs and their crossbreeds were used to produce Panxian ham, and their volatile flavor compounds were analyzed using GC-IMS and GC–MS. Multivariate statistical methods, including linear discriminant analysis effect size (LEfSe) and partial least squares discriminant analysis (PLS-DA), were applied to identify candidate discriminatory compounds among the groups. OAV/ROAV analysis was combined with partial least squares regression (PLSR) to further clarify the contribution of potential aroma compounds. These results can provide a scientific basis for raw-material optimization, flavor quality assessment, and precise process control of Panxian ham.

## 2 Materials and methods

### 2.1 Sample collection and preparation

Raw hind legs were obtained from three pig genetic-background groups: purebred Pingdi pigs (PDZ), Pingdi dam × Landrace sire crossbred pigs (CBZ), and Pingdi dam × Duroc sire crossbred pigs (DLK). All legs were obtained from castrated male pigs purchased in bulk by the processing enterprise from the same local production base. The pigs were managed under the routine conditions of the production base and received feed supplied uniformly by the base. In the winter of 2022, the pigs were slaughtered at an approximate live weight of 150 kg, and their legs were processed according to the standardized procedure of the enterprise (Mu et al., 2020). Specifically, the raw hind legs were chilled and aged at 4 °C for 24 h and subsequently salted with sea salt in cold storage at 4 °C. Salting was conducted in three stages over a total of 21 days. Salt was applied at 4% of the raw leg weight during the first stage and at 2% during each of the second and third stages, resulting in a total salt addition equivalent to 8% of the raw leg weight. After salting, residual salt and blood on the surface were removed by rinsing. The hams were then rested for 30 days at 10–15 °C and 75–80% relative humidity. Subsequently, they were hung in a fermentation and ripening room maintained at 20–22 °C and 65–70% relative humidity until fully ripened. The total processing and maturation period was 36 months. After maturation, three hams were randomly selected from each genetic-background group as independent biological replicates. After removal of the skin, the biceps femoris muscle was collected from each ham, vacuum-packaged, and stored at −80 °C until analysis.

### 2.2 Quantitative descriptive analysis of sensory attributes

Quantitative descriptive analysis was conducted with reference to GB/T 22210–2024. More than ten assessor candidates were initially recruited and screened based on their sensory sensitivity, odor-recognition ability, and capacity to discriminate among aroma attributes. Six assessors with the best overall performance were selected to form the trained sensory panel, comprising three males and three females aged 22–35 years. All selected assessors had professional and extensive experience in the sensory evaluation of Baijiu, a traditional Chinese distilled alcoholic beverage represented by Moutai, and were familiar with the recognition and description of complex aroma characteristics. Before the formal evaluation, the panelists were trained using standardized food-matrix references corresponding to each sensory descriptor. The reference materials were cooked pork for meaty aroma, pork fat for fatty aroma, yeast solution for fermented aroma, rose flowers for floral aroma, apples for fruity aroma, almonds for nutty aroma, button mushrooms (*Agaricus bisporus*) for mushroom aroma, and long-ripened dry-cured ham for aged aroma. Ham samples were thawed at 4 °C before assessment. After removing inedible surface parts, the biceps femoris muscle was cut into slices with a thickness of 1.5–2.0 mm. Approximately 10 g of each sample was placed in odorless white lidded cups and equilibrated at room temperature for 30 min. All samples were randomly coded and distributed to assessors. A 0–10 linear scale was adopted for scoring, where 0 represented undetectable odor and 10 represented extremely strong odor (Chen et al., 2023).

### 2.3 GC-IMS analysis

The analysis was conducted according to the method reported by Mu et al. (2026). Briefly, 2.0 g of minced ham sample was accurately weighed and transferred into a 20 mL headspace vial, which was then tightly sealed. The sample was incubated at 60 °C for 15 min with a stirring speed of 500 rpm. Subsequently, 500 μL of headspace gas was injected into the GC-IMS system (FlavourSpec®, Gesellschaft für Analytische Sensorsysteme mbH, Dortmund, Germany) at 85 °C in splitless mode. The GC system was equipped with an automatic headspace sampler (CTCPAL, CTC Analytics AG, Zwingen, Switzerland) and an MXT-5 capillary column (15 m × 0.53 mm × 1 μm, Restek, USA), with the column temperature maintained at 60 °C. High-purity nitrogen (99.999%) was used as the carrier gas. The flow rate program was set as follows: 2 mL/min from 0 to 2 min, then increased to 100 mL/min and held constant from 2 to 20 min. The temperature of the IMS drift tube was 45 °C, and the drift gas flow rate was 150 mL/min. Qualitative analysis was performed using VOCal software (v.1.0.7), Gallery Plot plug-in, NIST database and IMS database. Relative quantification was carried out based on peak volume.

### 2.4 GC–MS analysis

The detection method was modified slightly on the basis of Mu et al. (2025). In brief, 1.0 g of minced ham sample was placed into a 20 mL headspace vial, and 10 μL of internal standard solution (2-octanol, 3.1 mg/L) was added. A 120 μm DVB/CWR/PDMS solid-phase microextraction Arrow fiber was used for headspace extraction. The sample was equilibrated at 60 °C for 15 min and then adsorbed for 45 min. Afterwards, thermal desorption was performed in the GC–MS injection port at 250 °C for 1 min. An Agilent 6890 N-5975B GC–MS system coupled with an Agilent DB-WAX capillary column (60 m × 0.25 mm × 0.25 μm) was used for compound separation and identification. The oven temperature program was as follows: initial temperature of 40 °C held for 5 min, ramped to 200 °C at 6 °C/min, and maintained for 10 min. High-purity helium was used as the carrier gas at a constant flow rate of 1.0 mL/min with splitless injection mode. Mass spectrometry conditions: electron ionization (EI) source, electron energy of 70 eV, ion source temperature of 250 °C, and mass scanning range of 35–400 *m*/*z*. Mass spectral data were matched with the NIST 23 database, and only compounds with a similarity index above 800 were retained. Semi-quantification was performed using 2-octanol as the internal standard. For each 1.0 g ham sample, 10 μL of 2-octanol solution at 3.1 mg/L was added; the concentration of each volatile compound was estimated according to:

$$C_{x}=\frac{M_{x}}{M_{i}}\times \frac{C_{i}V_{i}}{m_{s}}\times \frac{1}{{\mathrm{RF}}_{x}}$$

where *C*_*x*_  is the semi-quantified concentration expressed as mg/kg 2-octanol equivalents, *M*_*x*_  and *M*_*i*_ are the peak areas of the target compound and internal standard, respectively, *C*_*i*_ is the concentration of the internal standard, *V*_*i*_ is the volume of internal-standard solution added, *m*_*s*_ is the sample mass, and *RF*_*x*_  is the relative response factor. Because compound-specific response factors were not determined, *RF*_*x*_ was assumed to be 1. Thus, the reported concentrations should be interpreted as semi-quantitative 2-octanol-equivalent values rather than absolute concentrations.

### 2.5 ROAV analysis

The ROAV method was applied to evaluate aroma-active compounds based on the relative content data obtained by GC-IMS. The compound contributing the most to sample flavor was defined with a ROAV value of 100, and the ROAV values of other components were calculated accordingly (Liu et al., 2009). The formula was as follows:

$$\mathrm{ROAV}=100\times \frac{C_{i}}{C_{\max}}\times \frac{T_{\max}}{T_{i}}$$

where *C*_*i*_ is the relative content (%) of a single volatile component; *T*_*i*_ is olfactory threshold (mg/kg) of the component; *C*_*max*_ and *T*_*max*_ represent the relative content (%) and olfactory threshold (mg/kg) of the reference component with the maximum flavor contribution, respectively.

### 2.6 OAV analysis

OAV was calculated using the semi-quantitative data acquired by GC–MS to assess aroma contribution of volatile compounds (Xiao et al., 2024). The calculation formula of OAV was as follows:

$$\mathrm{OAV}=\frac{C_{i}}{T_{i}}$$

where *C*_*i*_ is the content (mg/kg) of a volatile component, and *T*_*i*_ is its olfactory threshold (mg/kg).

### 2.7 Statistical analysis

All experiments were conducted with at least three replicates, and experimental data were expressed as mean ± standard deviation. One-way analysis of variance (ANOVA) was performed using SPSS 24.0 software, and differences with *p* < 0.05 were considered statistically significant. LEfSe with a threshold of linear discriminant analysis (LDA) > 3.0 and *p* < 0.05 was conducted through an open access pipeline (http://huttenhower.sph.harvard.edu/galaxy/). Principal component analysis (PCA), PLS-DA, and PLSR were carried out using SIMCA 14.1 software (Umetrics, Sweden). Each of the nine ham samples had one corresponding chemical profile and one sensory profile obtained from the trained panel. Thus, nine independent observations were entered into the PLSR model, with volatile compounds as the X variables and sensory attributes as the Y variables.

## 3 Results and discussion

### 3.1 Sensory characteristics of dry-cured hams from different pig breeds

Quantitative descriptive analysis (QDA) was adopted to systematically evaluate the sensory attributes of the three types of dry-cured hams, and the results were presented as mean ± standard deviation across the six trained assessors (Fig. 1). All samples presented typical aroma characteristics of dry-cured ham, and no significant differences were found in meaty, fermented, floral, and nutty aromas among the groups (*p* > 0.05). The scores of meaty aroma and nutty aroma followed the order of CBZ > DLK > PDZ, while fermented aroma and floral aroma showed an opposite trend. In comparison, CBZ and DLK hams had significantly higher scores for fatty and mushroom aromas than PDZ ham (*p* < 0.001), indicating that aromas related to lipid oxidation were more prominent in crossbred ham products. PDZ ham obtained a higher score for fruity aroma (*p* < 0.05), suggesting that aromatic compounds and esters contributed greatly to its sweet and floral notes. In addition, DLK ham had the highest score of aged aroma, which was significantly higher than that of PDZ ham (*p* < 0.05), demonstrating its more intense mature flavor. These results confirmed that pig breed exerted a remarkable effect on the flavor phenotypes of dry-cured ham, highlighting the need for a detailed analysis of the chemical basis.

**Fig. 1:**
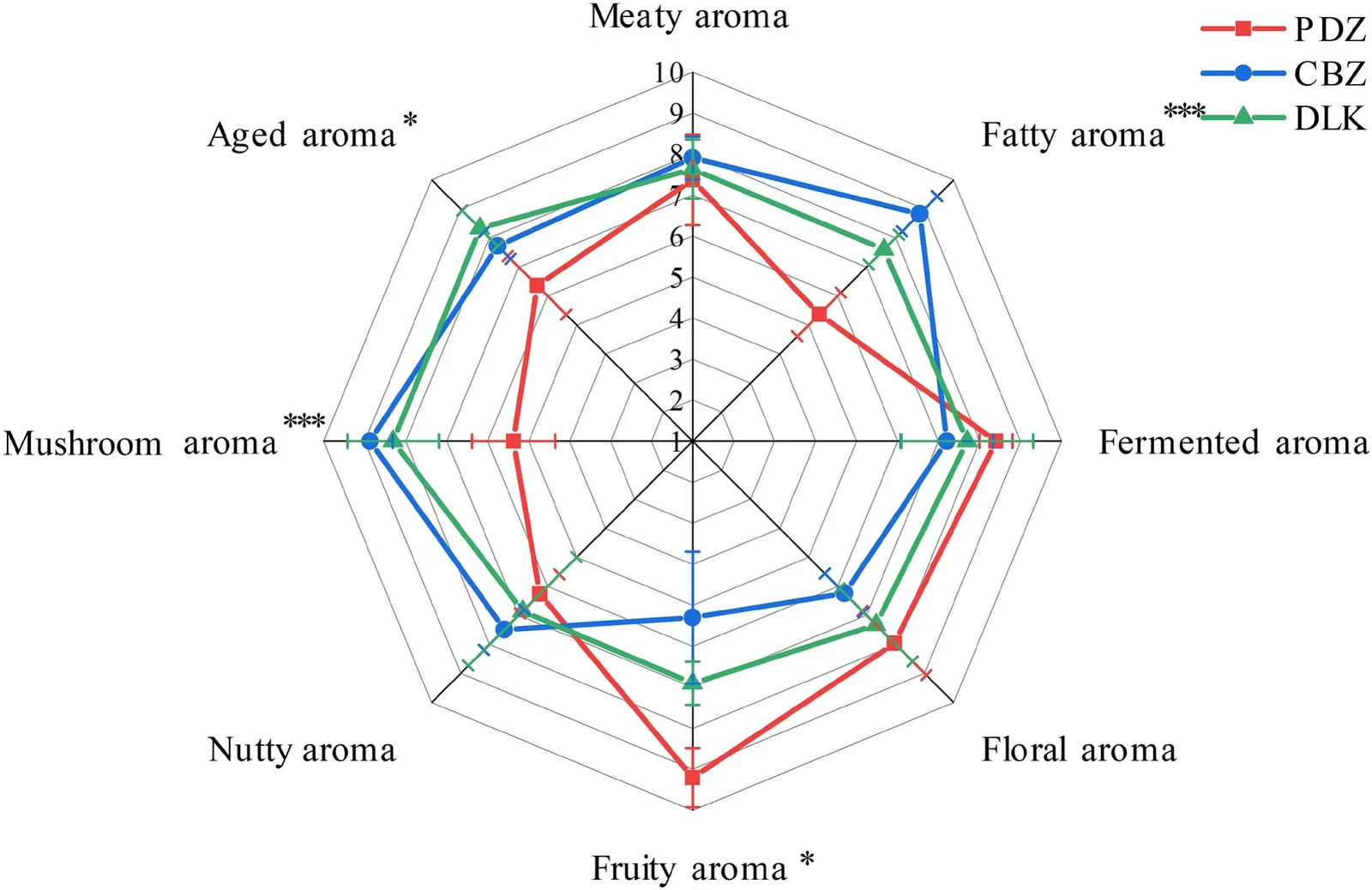
Quantitative descriptive analysis of sensory attributes of three types of dry-cured hams. *, *p* < 0.05; ***, *p* < 0.001. PDZ, purebred Pingdi pigs; CBZ, Pingdi dam × Landrace sire crossbred pigs; DLK, Pingdi dam × Duroc sire crossbred pigs.

### 3.2 Analysis of volatile compounds by GC-IMS

#### 3.2.1 Overall profile and classification of volatile compounds

GC-IMS was used to further explore the differences in volatile compounds among the three types of dry-cured ham. The three-dimensional and two-dimensional GC-IMS spectra illustrated the drift time, retention time, and ion signal intensity of the detected compounds (Fig. 2A-B). A total of 77 volatile compounds were identified, including 15 ketones, 13 aldehydes, 13 alcohols, 7 esters, 5 sulfur-containing compounds, 3 nitrogen-containing compounds, 3 hydrocarbons, 2 acids, 2 ethers, 1 furan, and 1 other compound (Table S1). Suffixes M (monomer) and D (dimer) were used to distinguish monomeric and dimeric forms of detected compounds. The PCA score plot showed obvious separation of flavor profiles among ham samples from different breeds (PERMANOVA: *p* = 0.0045), which verified that pig genetic background profoundly affected the overall volatile flavor profile of ham (Savić et al., 2025).

**Fig. 2:**
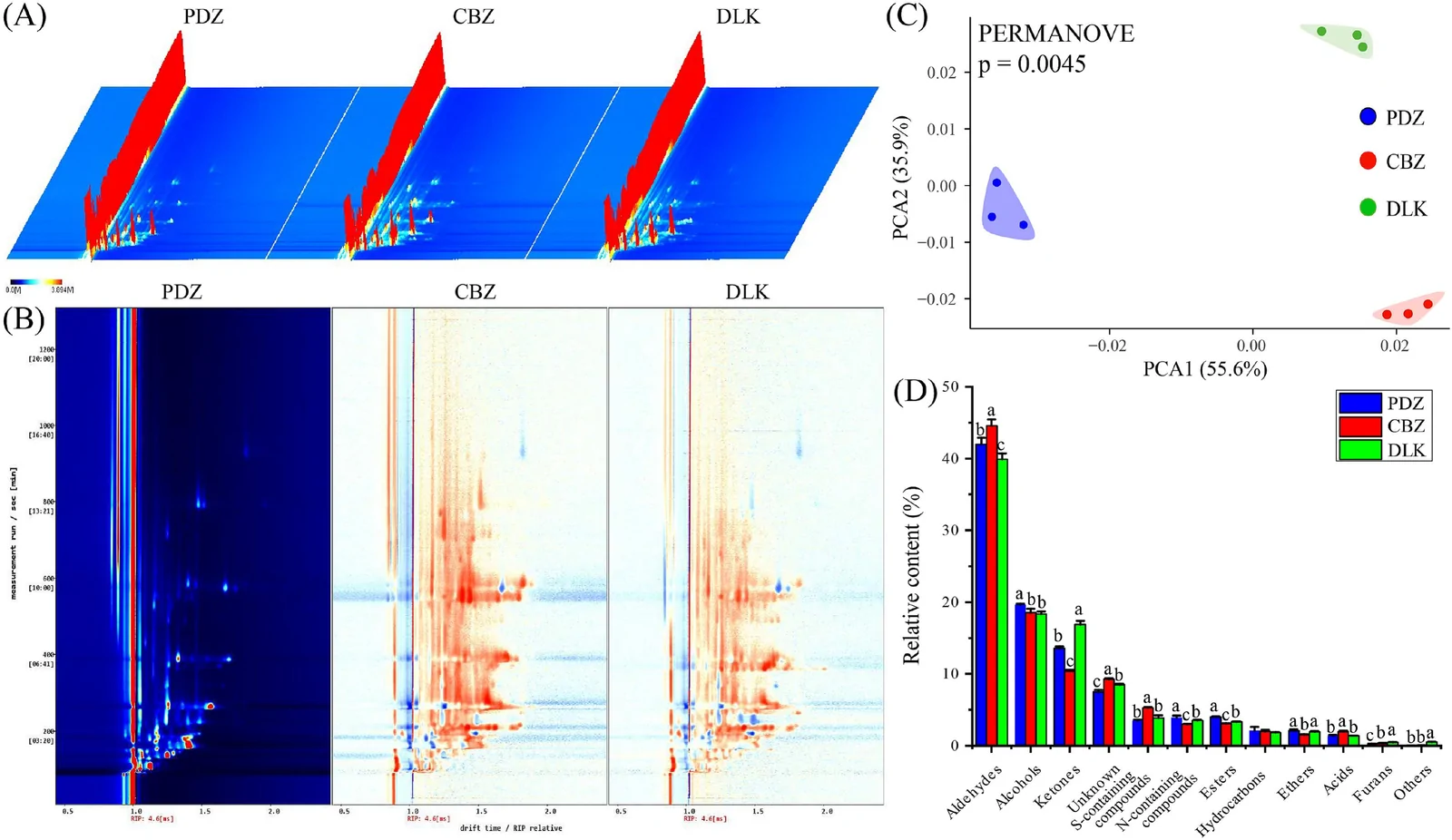
Gas chromatography–ion mobility spectrometry (GC-IMS) analysis of three types of dry-cured hams. Three-dimensional and two-dimensional spectra of all samples (A-B), principal component analysis (PCA) score plot based on all compounds (C), and differences in the relative abundances of compound classes among the three types of dry-cured hams (D). Different letters for the same type of compounds represent significant differences (*p* < 0.05). PDZ, purebred Pingdi pigs; CBZ, Pingdi dam × Landrace sire crossbred pigs; DLK, Pingdi dam × Duroc sire crossbred pigs.

Aldehydes were the most abundant category of compounds in all samples, accounting for 42.01%, 44.56%, and 39.90% in PDZ, CBZ, and DLK ham, respectively (Fig. 2D). Hexanal (15.79–17.34%) and 3-methylbutanal (12.79–16.92%) were the predominant aldehydes (Table S1). Our previous study on flavor changes during natural fermentation of Panxian ham revealed that aldehydes accounted for 25.20%–29.40% of total volatile compounds, among which hexanal and 3-methylbutanal were the most abundant linear aldehyde and branched-chain aldehyde, respectively (Mu et al., 2026). Studies on Jinhua ham and Nuodeng ham also proved that aldehydes dominated in mature ham and acted as key fingerprint components for distinguishing ham samples with different qualities (Li et al., 2022; Li et al., 2024). Aldehydes are mainly derived from the oxidative degradation of unsaturated fatty acids, and they are regarded as core contributors to the characteristic flavors of dry-cured ham such as lipid aroma and grassy notes due to their low olfactory thresholds (Chen et al., 2023; Liu et al., 2009).

Ketones (especially methyl ketones) are also important aroma contributors in dry-cured ham and are generally produced via lipid oxidation or amino acid degradation, endowing fruity, milky, and caramel flavors (Mu et al., 2026). In this study, the relative content of ketones in DLK ham (16.88%) was significantly higher than that in PDZ (13.57%) and CBZ (10.38%). 2-Butanone (2.86–5.75%), 2-pentanone-D (1.76–3.43%) and 2-heptanone-M (1.30–3.36%) were the dominant ketones in all groups. A high abundance of ketones was also reported in GC-IMS analysis of Iberian ham, and dominant ketones including 2-butanone and 2-heptanone were crucial to the aroma quality of high-grade ham (Rodríguez-Hernández et al., 2025). Alcohols maintained a relatively high abundance in dry-cured ham. The relative content of alcohols in PDZ ham (19.65%) was slightly higher than that in CBZ (18.57%) and DLK (18.35%), with 2-methyl-2-propanol (7.37–10.84%) as the main component. Alcohols are mainly generated from lipid oxidation or microbial reduction of carbonyl compounds, presenting fresh, woody, or mushroom-like aromas (Li et al., 2024). However, their direct contribution to overall flavor is weaker than aldehydes and ketones due to relatively high odor thresholds.

In addition, CBZ contained the highest levels of sulfur-containing compounds (5.28%) and acids (1.98%). By contrast, PDZ was enriched with esters (4.02%) and nitrogen-containing compounds (3.93%), and DLK had slightly higher levels of furans (0.48%) and other compounds (0.53%) (Table S1). Sulfur-containing compounds (e.g., dipropyl disulfide and furfuryl methyl sulfide), which have low odor thresholds, are formed through Strecker degradation of sulfur-containing amino acids and are important sources of meaty and onion-like flavors (Chen et al., 2023). Overall, pig breed significantly altered the composition of volatile flavor compounds in dry-cured ham. CBZ and DLK were characterized by a high abundance of aldehydes and ketones, respectively, while PDZ ham showed advantages in the accumulation of alcohols and esters.

#### 3.2.2 Identification of candidate discriminatory compounds via LEfSe

A Gallery Plot was used to establish volatile fingerprints to intuitively compare the relative content differences of volatile compounds among the three ham samples. Each row represented a single sample and each column represented one compound; the color depth of spots reflected the concentration of corresponding compounds. The fingerprint spectra showed both common and unique characteristics among the three hams (Fig. 3A). Hence, LEfSe analysis (LDA > 3, *p* < 0.05) was applied to screen putative breed-associated volatiles. A total of 29 candidate discriminatory compounds including 5 categories and 24 individual compounds were identified (Fig. 3B-C).

**Fig. 3:**
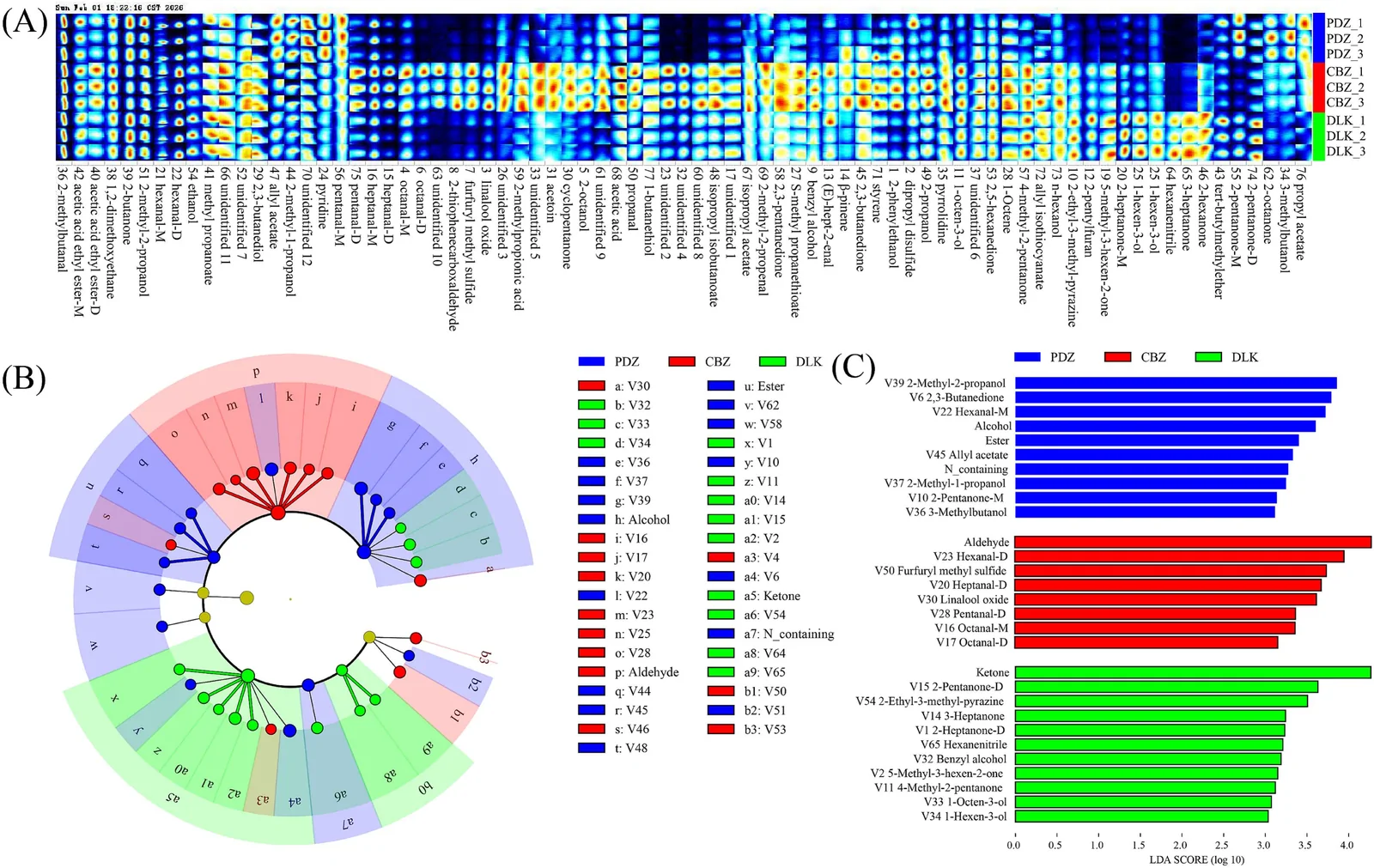
Peak intensity profiles of volatile compounds detected by gas chromatography–ion mobility spectrometry (GC-IMS) (A) and differential compounds identified by linear discriminant analysis effect size (LEfSe) analysis (B—C). PDZ, purebred Pingdi pigs; CBZ, Pingdi dam × Landrace sire crossbred pigs; DLK, Pingdi dam × Duroc sire crossbred pigs.

The candidate discriminatory compounds significantly enriched in PDZ were mainly alcohols and esters, such as 2-methyl-2-propanol, 2-methyl-1-propanol, 3-methylbutanol and allyl acetate. The relative abundance of alcohols in traditional Panxian ham was also higher than that in Jinhua ham and Xuanwei ham (Mu et al., 2026), which confirmed the superiority of PDZ ham in alcohol metabolism. Among them, 2-methyl-2-propanol is dominant in dry-cured ham and has been identified as a flavor marker of traditional Panxian ham (Jia et al., 2024; Mu et al., 2026), accounting for 10.12% of PDZ ham. Together with 3-methylbutanol (1.02%) and 2-methyl-1-propanol (1.16%), they might provide fresh wine-like and fermented aromas for PDZ ham (Fig. 1).

The candidate discriminatory compounds of CBZ were mainly aldehydes, including hexanal-D, heptanal-D, pentanal-D, octanal-M, linalool oxide and furfuryl methyl sulfide. Consistent with numerous previous studies (Li et al., 2022; Rodríguez-Hernández et al., 2025), hexanal derived from linoleic acid oxidation was one of the most abundant aldehydes in ham. The relative content of hexanal-D in CBZ reached 11.09%, acting as the core contributor to fatty aroma (Fig. 1). Moreover, linear aldehydes including heptanal-D (2.76%), pentanal-D (2.35%) and octanal-M (1.75%) further layered nutty and citrus aromas for CBZ.

Ketones were the preferred candidate differentiated compounds for DLK, such as 2-pentanone-D, 2-heptanone-D, 3-heptanone, 5-methyl-3-hexen-2-one and 4-methyl-2-pentanone. Among them, 2-pentanone and 2-heptanone have been defined as characteristic compounds of Jinhua ham and Yunnan ham (Li et al., 2022; Yang, He, et al., 2023), and their proportions in the DLK were 3.38% and 0.75%. Moreover, 4-methyl-2-pentanone was confirmed as a vital characteristic flavor compound distinguishing premium-grade Jinhua ham from ordinary grades (Zhao et al., 2014).

### 3.3 Analysis of volatile compounds by GC–MS

#### 3.3.1 Overall profile and classification of volatile compounds

GC–MS was adopted for a comprehensive analysis of the effects of breed on ham flavor quality. A total of 58 compounds were identified, covering 16 aldehydes, 11 esters, 9 acids, 6 alcohols, 3 nitrogen-containing compounds, 3 furans, 3 hydrocarbons, 2 ketones, 2 phenols, 2 sulfur-containing compounds and 1 other compound (Table S2). The cluster heatmap and PCA score plot revealed obvious differences in the types and contents of volatile compounds among the three groups (PERMANOVA: *p* = 0.0028) (Fig. 4A-B). Notably, only nine volatile compounds were commonly identified by both GC-IMS and GC–MS (Fig. 4C), suggesting that the two platforms captured partially overlapping but distinct portions of the volatile profile. This limited overlap originates from combined differences in chromatographic column selectivity, ionization mechanisms and detector response characteristics. In this study, GC-IMS was equipped with a non-polar MXT-5 chromatographic column coupled with atmospheric-pressure ion-molecule reaction ionization. This configuration delivers extraordinary sensitivity for highly volatile small-molecule polar analytes including low-boiling aldehydes, ketones and short-chain alcohols, yet exhibits poor response toward larger semi-volatile molecules (Arroyo-Manzanares et al., 2018). By comparison, GC–MS adopted a polar DB-WAX capillary column and electron-impact (EI) vacuum ionization (Quintanilla-Casas et al., 2025). The polar stationary phase favors retention and separation of medium-to-long-chain semi-volatile substances; paired with comprehensive standard mass spectral libraries, GC–MS enables reliable identification of long-chain fatty acids and long-chain esters that are hardly detected by GC-IMS. Beyond separation and ionization discrepancies, the two platforms also differ in data nature: GC-IMS mainly generates relative fingerprint-level peak-volume signals, whereas GC–MS provides library-validated compound-specific mass-spectral information. Therefore, various high-molecular-weight compounds were successfully detected by GC–MS, while abundant low-molecular-weight compounds were identified by GC-IMS.

**Fig. 4:**
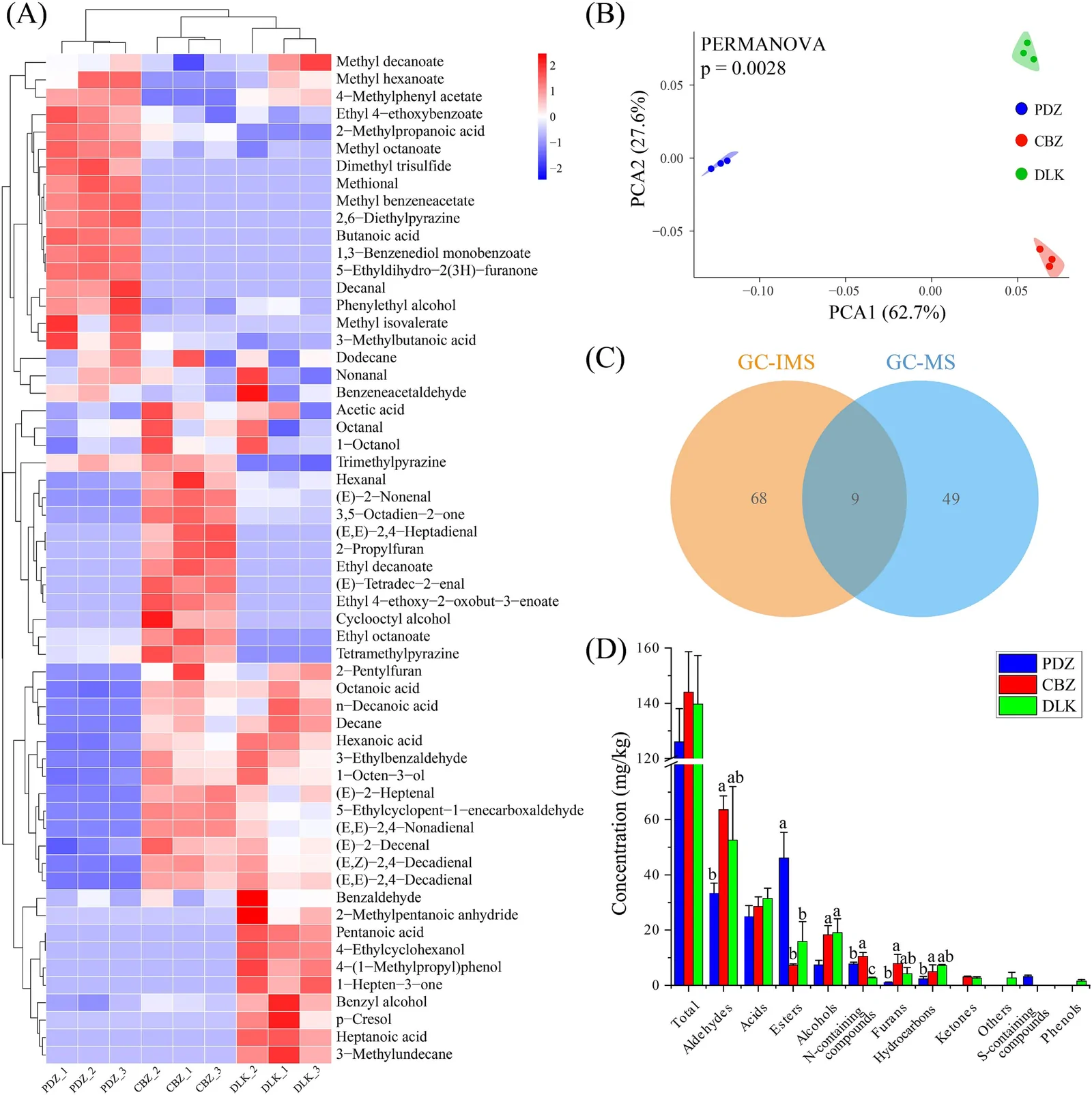
Gas chromatography–mass spectrometry (GC–MS) analysis of three types of dry-cured hams. Cluster heatmap of all volatile compounds (A), principal component analysis (PCA) score plot based on all detected compounds (B), Venn diagram of volatile compounds detected by gas chromatography–ion mobility spectrometry (GC-IMS) and GC–MS (C), and differences in the content compound classes among the three types of dry-cured hams (D). Different letters for the same type of compounds represent significant differences (*p* < 0.05). PDZ, purebred Pingdi pigs; CBZ, Pingdi dam × Landrace sire crossbred pigs; DLK, Pingdi dam × Duroc sire crossbred pigs.

The total content of volatile compounds was the highest in CBZ (144.02 mg/kg), followed by DLK (139.67 mg/kg) and PDZ (125.99 mg/kg), but there was no significant difference among them (Fig. 4D). Consistent with GC-IMS results, aldehydes were the most abundant components in all samples. The content of aldehydes in CBZ was up to 63.59 mg/kg, significantly higher than that in DLK (52.54 mg/kg) and PDZ (33.36 mg/kg) (*p* < 0.05). Hexanal had the highest content in CBZ (18.43 mg/kg), while phenylacetaldehyde was the dominant aldehyde in PDZ (12.16 mg/kg) and DLK (12.34 mg/kg) (Table S2). Hexanal is formed by oxidative degradation of n-6 unsaturated fatty acids (such as linoleic acid), serving as the most abundant linear aldehyde in meat products and contributing fatty and grassy aromas (Calkins & Hodgen, 2007). Phenylacetaldehyde is mainly produced by Strecker degradation of phenylalanine. As a characteristic floral component in dry-cured ham, it can neutralize greasy taste and enhance the sweet and mellow notes of meat (Resconi et al., 2013). These results suggested that lipid oxidation was more intense in CBZ, while protein degradation and amino acid transformation were more extensive in PDZ and DLK.

Acids were the second largest category of flavor components. DLK had the highest content (31.40 mg/kg), followed by CBZ (28.53 mg/kg) and PDZ (24.91 mg/kg), with hexanoic acid (6.27–21.52 mg/kg) as the main compound. Acids are mainly derived from lipid hydrolysis and microbial metabolism. Appropriate contents of short-chain fatty acids (e.g., hexanoic acid, 3-methylbutanoic acid) bring sour, cheesy and fermented flavors, while excessive acids may cause pungent sourness and sweaty off-flavor (Flores, 2018). In accordance with GC-IMS results, the ester content in PDZ (46.09 mg/kg) was far higher than that in DLK (15.86 mg/kg) and CBZ (7.22 mg/kg), especially methyl 3-methylbutanoate (18.44 mg/kg) and methyl hexanoate (17.59 mg/kg). Esters are generated via esterification between fatty acids and alcohols, providing fruity and floral aromas for ham (Yang, He, et al., 2023). The alcohol contents of DLK (19.07 mg/kg) and CBZ (18.27 mg/kg) were higher than those of PDZ (7.38 mg/kg), with 1-octen-3-ol as the main compound. It is a representative alcohol compound with a typical mushroom flavor and has been confirmed as a crucial flavor compound in various dry-cured meat products (Petričević et al., 2018; Wu et al., 2021). These differences might explain the highest scores for fruity and floral aromas and the lowest score for mushroom aroma in PDZ (Fig. 1).

Nitrogen-containing compounds (namely pyrazines) had the highest content in CBZ (10.53 mg/kg), followed by PDZ (7.71 mg/kg) and DLK (2.69 mg/kg), and trimethylpyrazine was the dominant component. This result was opposite to that of GC-IMS, which was probably due to the stronger detection capability of GC–MS for pyrazines (Li et al., 2023). As major products of the Maillard reaction and microbial metabolism, pyrazines accumulate largely in the late maturation stage of ham and produce roasted and nutty aromas (Du et al., 2025; Jia et al., 2024). Furans also reached the highest level in CBZ ham (7.84 mg/kg), and 2-pentylfuran was the main contributor. Notably, phenols (1.49 mg/kg) including p-cresol and 4-(1-methylpropyl)phenol were only detected in DLK ham. Since no smoking process was applied during Panxian ham production, these phenols were mostly derived from tyrosine degradation, contributing smoky and herbal aromas (Mu et al., 2020; Ruiz et al., 2002). In contrast, sulfur-containing compounds (3.09 mg/kg) such as dimethyl trisulfide and 3-(methylthio)propanal were solely found in PDZ ham. These compounds originated from the degradation of sulfur-containing amino acids, presenting meaty and onion-like flavors (Chen et al., 2023).

#### 3.3.2 Identification of candidate discriminatory compounds via PLS-DA

The PLS-DA model was established based on GC–MS data to further screen putative breed-associated volatiles. Seven-fold cross-validation was implemented for model evaluation, and the model parameters R2X, R2Y and Q2 were 0.869, 0.990 and 0.945, respectively (Fig. 5A). Meanwhile, a permutation test with 200 random permutations yielded an intercept of −0.202 for Q2, indicating no apparent over-fitting based on internal validation (Fig. 5B). It should be noted that this multi-class PLS-DA was built with only three biological replicates per group; although cross-validation and permutation test supported its performance, the predictive generalization to fully independent external samples remain untested.

**Fig. 5:**
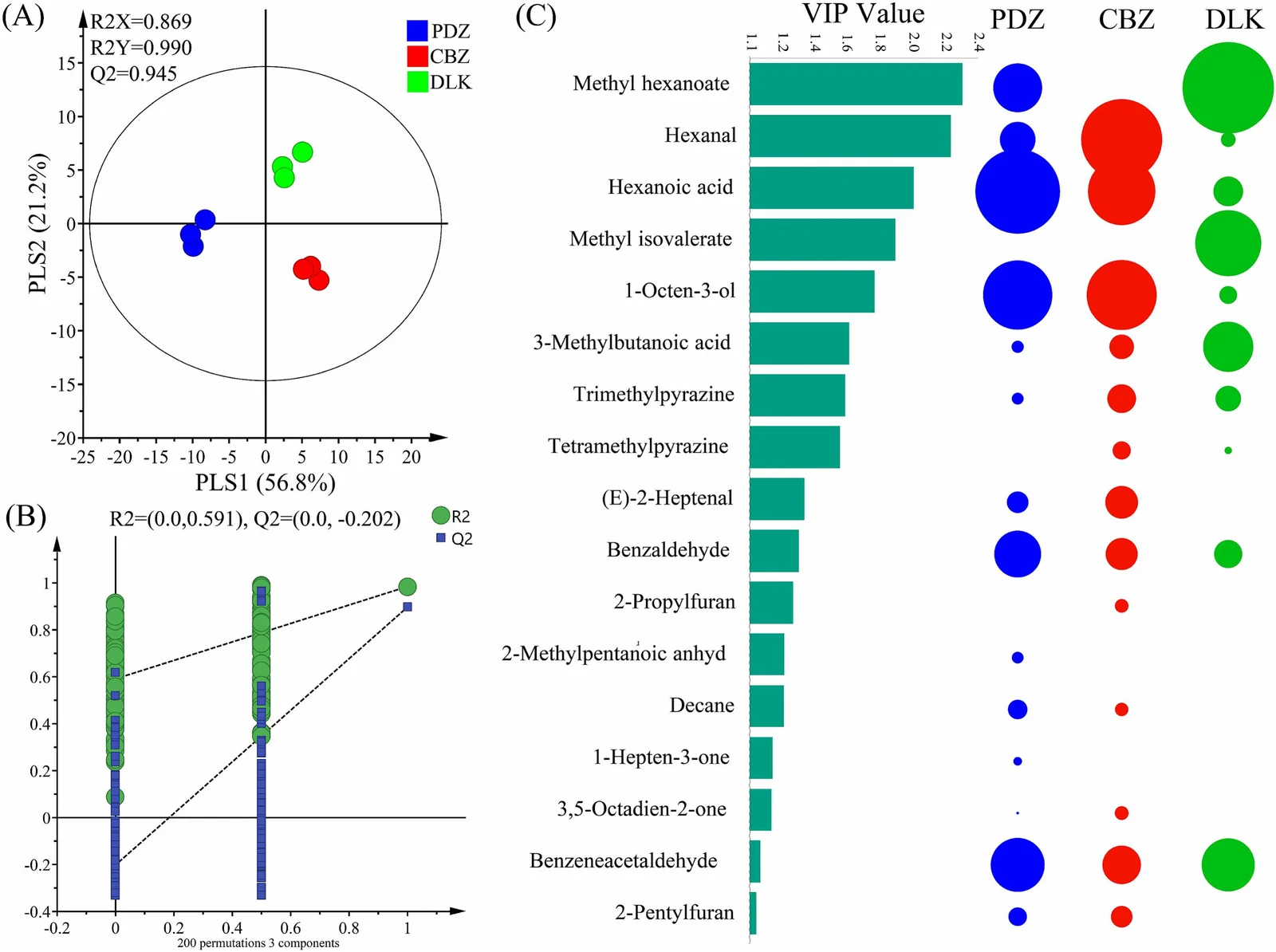
Partial least squares discriminant analysis (PLS-DA) score plot based on all compounds detected by gas chromatography–mass spectrometry (GC–MS) (A), 200 permutation tests of the PLS-DA model (B), and variable importance in projection (VIP) scores of differential compounds among the three types of dry-cured hams (C). PDZ, purebred Pingdi pigs; CBZ, Pingdi dam × Landrace sire crossbred pigs; DLK, Pingdi dam × Duroc sire crossbred pigs.

A total of 17 candidate discriminatory compounds were screened with variable importance in projection (VIP) > 1 (Fig. 5C). Among them, methyl hexanoate and methyl 3-methylbutanoate were enriched in PDZ. Two plausible explanations may account for this observation: (i) higher intramuscular fat content of raw pork may facilitate endogenous lipid decomposition (Wang et al., 2025); (ii) fermentative microorganisms might exert enhanced synergistic esterase activity (Zheng et al., 2020). The content of 3-methylbutanoic acid in PDZ ham (11.48 mg/kg) was also higher than that in CBZ (5.52 mg/kg) and DLK (2.78 mg/kg). This compound is derived from the Strecker degradation of leucine or metabolism by lactic acid bacteria, and it is an essential component of fermented flavor in dry-cured ham (Petričević et al., 2018).

Candidate discriminatory compounds with high VIP values in CBZ included hexanal, (E)-2-heptenal, 2-propylfuran, trimethylpyrazine, tetramethylpyrazine, and 3,5-octadien-2-one. Hexanal (18.44 mg/kg) and (E)-2-heptenal (7.39 mg/kg) are common lipid oxidation products in dry-cured meat products and contribute to prominent lipid aroma (Chen et al., 2023). Trimethylpyrazine (6.46 mg/kg) and tetramethylpyrazine (4.06 mg/kg) are generated from the Maillard reaction and microbial metabolism, providing roasted and nutty aromas (Li, Liu, et al., 2025).

For the DLK group, candidate discriminatory compounds included hexanoic acid, benzaldehyde, decane, and 1-hepten-3-one. Hexanoic acid (19.37 mg/kg), derived from lipid oxidation and microbial metabolism, enhanced the sour flavor of ham, while benzaldehyde (10.72 mg/kg) from phenylalanine degradation contributed almond and nutty aromas (Flores, 2018). In addition, 1-hepten-3-one (1.95 mg/kg) was exclusively detected in DLK and imparted an intense mushroom aroma (Wu et al., 2026).

### 3.4 Comprehensive analysis of the effects of candidate discriminatory compounds on overall flavor

The actual contribution of volatile compounds to the overall flavor of dry-cured ham depends not only on their relative or absolute contents, but also on their odor thresholds, odor characteristics, and the synergistic or masking effects among different compounds (Li, Shi, et al., 2025). Therefore, analyzing merely the contents of candidate discriminatory compounds cannot accurately clarify the flavor-driving factors of dry-cured hams from different pig breeds. In this study, ROAV (based on GC-IMS data) and OAV (based on GC–MS data) were combined to screen potential aroma-active compounds. PLSR was further used to analyze the correlation between candidate compounds and sensory attributes, so as to explore the chemical basis for flavor differences among hams from different breeds (Xu et al., 2025). Notably, because the release, partitioning, and sensory perception of volatile compounds are strongly matrix-dependent, odor thresholds determined in oil were preferentially selected when available. Compared with thresholds measured in water, oil-based thresholds may better approximate the behavior of hydrophobic volatile compounds in the lipid-rich environment of dry-cured ham (Chen et al., 2023). For compounds for which reliable oil-based thresholds were unavailable in the literature (van Gemert, 2011), thresholds determined in water were used as an alternative to avoid excluding potentially important aroma-active compounds from the analysis. Although this might affect the absolute values and comparability of the calculated OAV and ROAV values, these indicators are mainly used as semi-quantitative screening tools rather than as precise measures of their sensory contributions.

#### 3.4.1 ROAV analysis based on GC-IMS data

ROAV evaluates the relative flavor contribution of volatile compounds by taking the component with the maximum overall aroma contribution as the reference. Compounds with ROAV≥1 are defined as main aroma-active compounds, while those with 0.1 ≤ ROAV<1 can regulate the overall flavor profile via aroma modification, superposition, and synergistic effects (Liu et al., 2009).

A total of 12 candidate discriminatory compounds with potential flavor contributions were screened by GC-IMS-based ROAV analysis (Fig. 6A & Table S3). 1-Octen-3-ol had the highest ROAV values in all three ham samples, namely 69.57 (PDZ), 91.99 (CBZ), and 100.00 (DLK), indicating that it was the common core aroma-active compound of the three dry-cured hams. It is mainly produced by enzymatic oxidation or auto-oxidation of polyunsaturated fatty acids such as linoleic acid, with typical mushroom, earthy, and fatty aromas (Xiao et al., 2024). PLSR results showed a strong correlation between this compound and mushroom and fatty aromas (Fig. 6C), suggesting that it not only had high relative odor activity but also may contribute to the mature aroma of ham at the sensory level. Similar results have been reported in traditional dry-cured meat products such as Iberian ham and Jinhua ham, suggesting that 1-octen-3-ol is a potential aroma-active compound formed during ham maturation (Arroyo-Manzanares et al., 2018; Chen et al., 2023).

**Fig. 6:**
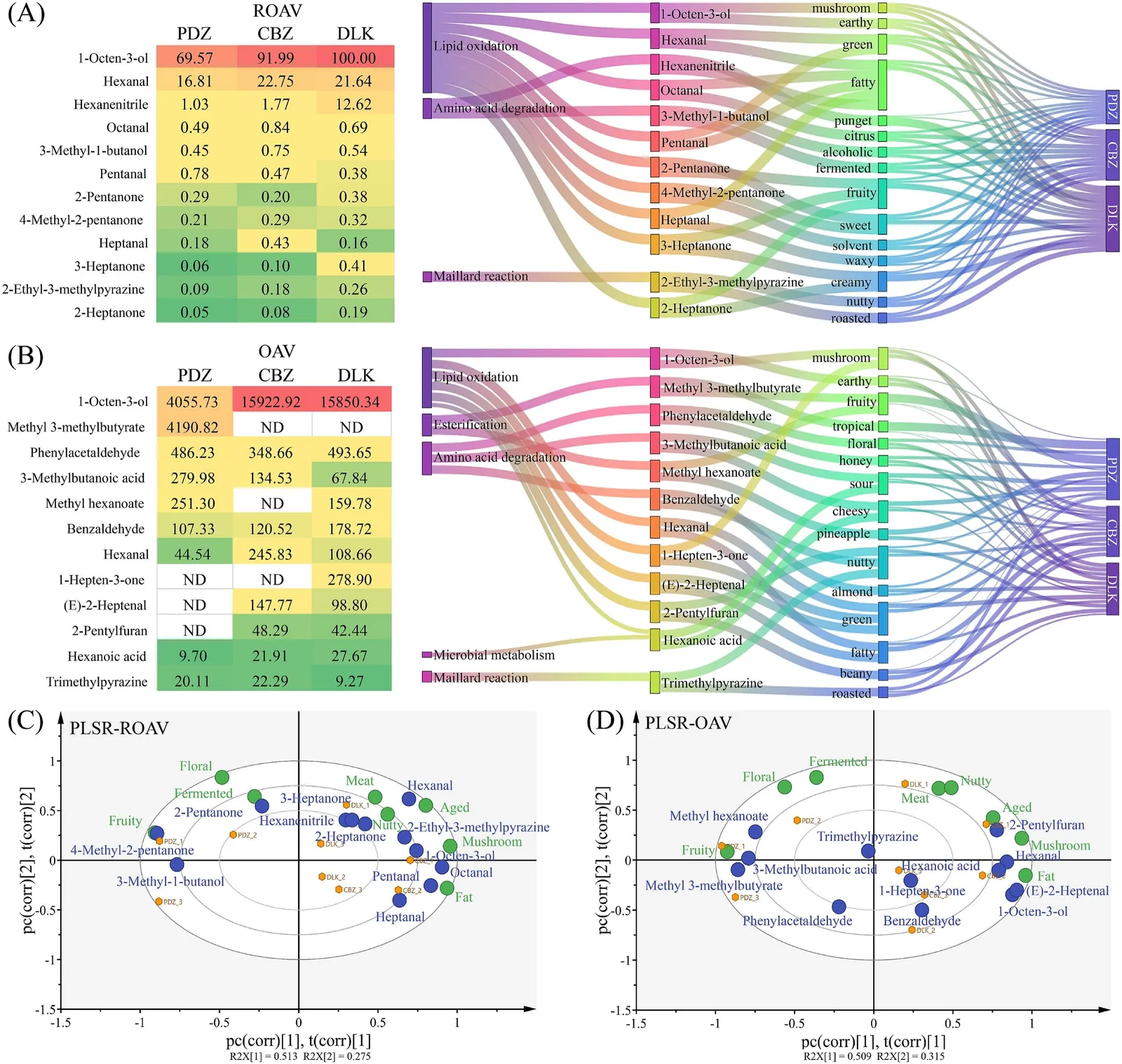
Potential aroma-active compounds identified by relative odor activity value (ROAV) and odor activity value (OAV) analysis and their contribution to the flavor characteristics of three types of dry-cured hams (A-B), and partial least squares regression (PLSR) analysis of these aroma-active compounds and the sensory attributes of dry-cured hams (C—D). PDZ, purebred Pingdi pigs; CBZ, Pingdi dam × Landrace sire crossbred pigs; DLK, Pingdi dam × Duroc sire crossbred pigs.

Hexanal was another potential aroma compound in all samples, with an ROAV of 16.81 (PDZ), 22.75 (CBZ), and 21.64 (DLK). It is mainly derived from linoleic acid oxidation, presenting grassy and fresh notes at low concentrations and a distinct lipid aroma at high concentrations (Lorenzo et al., 2013). The high ROAV of hexanal in all samples indicated that lipid oxidation was the common foundation for the formation of mature flavor of dry-cured hams from different breeds. Notably, CBZ had the highest ROAV of hexanal, and PLSR results showed that hexanal may be correlated with fatty aroma (Fig. 6C), supporting the higher fatty aroma score of CBZ in sensory evaluation (Fig. 1). It was further confirmed that hexanal can be used as an important indicator to reflect lipid oxidation intensity and fatty aroma formation (Liu et al., 2020; Wang et al., 2018).

Hexanenitrile was also classified as a main flavor component, showing a significantly higher ROAV of 12.62 in DLK than that in CBZ (1.77) and PDZ (1.03) (Fig. 6A). Compared with typical lipid oxidation products, nitriles have been rarely reported in dry-cured ham. They are usually related to the degradation of nitrogen-containing amino acids or metabolism of specific microorganisms. Although direct research on the flavor function of hexanenitrile in dry-cured ham is insufficient, existing studies have found that nitriles are important chemical markers for distinguishing characteristic hams from different regions of China, implying that nitriles play a unique role in flavor formation of ham with specific raw materials or processing techniques (Li et al., 2021). PLSR analysis revealed that hexanenitrile may be associated with aged, fruity, and meaty aromas (Fig. 6C), suggesting its participation in the formation of complex flavor of DLK.

For compounds with ROAV ranging from 0.1 to 1, octanal and heptanal were mainly generated from fatty acid oxidation, with citrus, fatty, waxy, and green notes (Ruiz et al., 2002). Their relatively higher ROAV values (0.84 and 0.43) in CBZ ham further verified the stronger lipid oxidation in this group (Fig. 6A). 3-Methyl-1-butanol, derived from leucine degradation, has wine-like, fermented, and slightly pungent odors (Marušić et al., 2014). Its moderate ROAV (0.45–0.75) in all three groups indicated that amino acid degradation and microbial metabolism jointly participated in the formation of fermented aroma of all hams. Ketones including 2-pentanone, 4-methyl-2-pentanone, 3-heptanone, and 2-heptanone are closely related to fatty acid β-oxidation, microbial metabolism, and the long-term maturation process, generally presenting fruity, sweet, creamy and slight fatty aromas (Li et al., 2022; Zhao et al., 2016). Notably, 3-heptanone and 2-heptanone had the highest ROAV values (0.41 and 0.38) in DLK, and PLSR analysis showed that they may contribute to the aged, meaty, and nutty aromas (Fig. 6C). Previous studies have proven that ketones such as 2-heptanone and 2-nonanone are important compounds contributing to aged aroma in tea (Yang, Peng, et al., 2023). Although the ROAV values of these ketones were generally lower than 1, they might modify the mature flavor of DLK through aroma interactions with low thresholds and particularly contribute to the formation of aged aroma (Fig. 1).

#### 3.4.2 OAV analysis based on GC–MS data

Different from ROAV, which is calculated based on relative content, OAV analysis based on semi-quantitative data can more accurately identify potential aroma-active compounds with remarkable sensory contributions to overall flavor. Taking OAV ≥ 1 as the screening criterion, a total of 12 candidate compounds were identified (Fig. 6B & Table S4).

Consistent with ROAV results, 1-octen-3-ol was the most important aroma contributor. Its higher OAV values in CBZ (15,922.92) and DLK (15,850.34) contributed to a stronger mushroom aroma (Fig. 6D). The OAV of hexanal followed the order of CBZ (245.83) > DLK (108.66) > PDZ (44.54), which was highly consistent with ROAV results. (E)-2-heptenal and 2-pentylfuran were also enriched in CBZ, with OAV values up to 147.77 and 48.29, respectively (Fig. 6B). It has been reported that the content of hexanal and (E)-2-heptenal in dry-cured ham is closely associated with lipid oxidation intensity (Marušić et al., 2014; Petričević et al., 2018; Ruiz et al., 2002). Moreover, the total aldehyde content was the highest in CBZ and the lowest in PDZ (Fig. 4D), which indicated that CBZ may have developed prominent fatty aroma characteristics through more intense lipid oxidation (Fig. 1).

PDZ was characterized by remarkable contributions of esters and branched-chain fatty acids (Fig. 6B). Methyl 3-methylbutanoate was exclusively detected in PDZ with an extremely high OAV of 4190.82, ranking as the second most important aroma contributor after 1-octen-3-ol. Methyl hexanoate also had a high OAV of 251.30 in PDZ, while it was not detected in CBZ ham. Short-chain fatty acid esters are formed through esterification between fatty acids and alcohols under the catalysis of microbial esterases. With low thresholds and pleasant odors, they can significantly regulate overall flavor even at low concentrations. PLSR analysis showed that methyl 3-methylbutanoate and methyl hexanoate may be associated with fruity and floral aromas (Fig. 6D). In addition, 3-methylbutanoic acid was another important flavor contributor in PDZ, with an OAV of 279.98, approximately twice that of CBZ (134.53) and four times that of DLK (67.84). A high content of 3-methylbutanoic acid is regarded as a marker for the deep maturation of ham (Carrapiso et al., 2010). From the synchronous enrichment of the above-mentioned compounds, it is hypothesized that PDZ may possess unique characteristics in terms of fatty-acid composition, esterification activity, or relevant microbial metabolic pathways, consequently generating its typical flavor profile dominated by fruity, floral, and fermented aromas (Fig. 1).

In contrast, the potential aroma compounds of DLK showed greater diversity. Phenylacetaldehyde was an important common flavor component of all samples, but its highest OAV (493.65) was observed in DLK. As a typical Strecker aldehyde in dry-cured ham, phenylacetaldehyde has honey-like and floral aromas, and its high activity may relate to protein degradation and amino acid transformation (Flores, 2018). Benzaldehyde, derived from phenylalanine metabolism, also had the highest OAV (178.72) in DLK, indicating that protein degradation and microbial metabolism might be more active. Moreover, 1-hepten-3-one was only detected in DLK with an OAV of 278.90, contributing unique metallic and mushroom aromas (Wu et al., 2026). The high OAV of hexanoic acid (27.67) may partially offset the insufficient sour and fermented flavors of DLK to a certain extent. PLSR analysis showed that the correlation between these compounds and sensory attributes was relatively weak (Fig. 6D), which might explain the balanced aroma profile of DLK (Fig. 1).

## 4 Conclusions

This study provides a Panxian ham-specific characterization of the relationship between pig genetic background, volatile composition, and sensory attributes, demonstrating that their abundance patterns and sensory associations differed among purebred Pingdi, Pingdi × Landrace, and Pingdi × Duroc genetic backgrounds under the same production framework. PDZ may have stronger potential in esterification and microbial metabolism, resulting in intense floral, fruity, and fermented aromas. CBZ may experience intense lipid oxidation and the Maillard reaction, which established its sensory advantages in fatty, mushroom, and nutty aromas. DLK was characterized by higher levels of ketones, phenylacetaldehyde, hexanenitrile, and various compounds, resulting in a balanced flavor with a prominent aged aroma. From an industrial perspective, these findings indicate that pig genetic background can be used as an upstream factor for flavor-oriented raw-material selection in Panxian dry-cured ham production. The putative breed-associated volatiles identified in this study may help classify raw materials and predict the formation of fatty, mushroom, floral, and aged aroma attributes. Accordingly, producers may select pig genetic backgrounds associated with the desired flavor profile and use the candidate discriminatory compounds for batch evaluation and quality control. In addition, the identified compounds may serve as chemical indicators for monitoring the ripening process and for guiding future optimization of curing temperature, relative humidity, salt level, and ripening duration. Overall, although the aroma contributions of the identified compounds have not been validated by recombination and omission tests, integration of genetic background screening and volatile marker monitoring facilitates the production of Panxian ham with improved uniformity and enhanced flavor profile.

## CRediT authorship contribution statement

**Dong Li:** Writing – original draft, Investigation. **Ying Huang:** Writing – original draft, Visualization. **Jun Lv:** Investigation, Data curation. **Chi Zhao:** Supervision, Conceptualization. **Chunlin Zhang:** Supervision, Project administration. **Hong Zhang:** Writing – review & editing, Investigation. **Yu Mu:** Writing – review & editing, Supervision, Resources, Formal analysis, Conceptualization.

## Ethics declaration

Verbal informed consent to take part in the study and to publish the article was obtained from all participants or their legal representatives. Appropriate protocols to protect participants' rights and privacy were strictly followed. Verbal informed consent was obtained from all participants prior to their involvement. No personally identifiable information was collected or stored at any stage of the study to ensure privacy protection. All data presented in this manuscript were derived from the above ethically compliant procedures. The privacy rights of participants have been observed.

This study was performed in compliance with relevant laws, regulatory frameworks and guidelines where the research took place. The Human Ethics Committee of Moutai Institute granted an exemption. The authors provided the following explanation: This study involved the sensory evaluation of food products. According to the policy of the Human Ethics Committee of Moutai Institute, this type of research does not require formal ethical approval. All procedures were conducted in strict accordance with the Standard Guide for Protection of Respondents and Informed Consent for Sensory Evaluation Studies (ASTM International, E3314–21) and aligned with the principles of the Declaration of Helsinki.

## Declaration of competing interest

The authors declare that the research was conducted in the absence of any commercial or financial relationships that could be construed as a potential conflict of interest.

## Data Availability

Data will be made available on request.

## Appendix A Supplementary data

Table S1 Detailed information on volatile compounds detected by HS-GC-IMS. Table S2 Detailed information on volatile compounds detected by HS-SPME-GC-MS. Table S3 The ROAV results based on differential compounds identified by HS-GC-IMS. Table S4 The OAV results based on differential compounds identified by HS-SPME-GC-MS.
