# Supplementary material for: Elucidating the role of genetic background in shaping the flavor characteristics of Panxian dry-cured ham: A multidimensional approach integrating sensory evaluation, GC-IMS, and GC–MS
Source: Food Chem X. 2026 Oct 8;39:104595. doi: 10.1016/j.fochx.2026.104595 (PMC13677972; doi:10.1016/j.fochx.2026.104595)
Supplement: Supplementary file 1 [file mmc1.docx]

**Supplementary material**

**Elucidating the role of genetic background in shaping the flavor characteristics of Panxian dry-cured ham: A multidimensional approach integrating sensory evaluation, GC-IMS, and GC-MS**

**Dong Li^a,b,1^, Ying Huang^c,1^, Jun Lv^a,b^, Chi Zhao^a,b^, Chunlin Zhang^c^, Hong Zhang^a,b,*^, Yu Mu^a,b,*^**

^a^ School of Food Engineering, Moutai Institute, Renhuai, 564507, China;

^b^ Guizhou Engineering Research Center for Health-Functional Liquor Brewing Technology, Moutai Institute, Renhuai 564507, China;

^c^ School of Brewing Engineering, Moutai Institute, Renhuai, 564507, China.

**^1^ Both authors contributed equally to the manuscript**

*** The complete contact details of the corresponding author:**

Name: Hong Zhang; Yu Mu

E-mail: hongzhang1986@163.com; muyu@mtxy.edu.cn

Phone: +86-13765820409; +86-18164881025

Mailing address: School of Food Engineering, Moutai Institute, Renhuai, 564507, China.

**Table S1** Detailed information on volatile compounds detected by HS-GC-IMS

| **Compounds** | **Relative content (%)** | | | | | | | | |
| --- | --- | --- | --- | --- | --- | --- | --- | --- | --- |
|  | **PDZ-1** | **PDZ-2** | **PDZ-3** | **CBZ-1** | **CBZ-2** | **CBZ-3** | **DLK-1** | **DLK-2** | **DLK-3** |
| ***Ketones*** | **13.85** | **13.45** | **13.40** | **10.32** | **10.57** | **10.27** | **16.29** | **17.28** | **17.05** |
| 2-heptanone-D | 0.20 | 0.19 | 0.19 | 0.33 | 0.28 | 0.32 | 0.70 | 0.74 | 0.80 |
| 5-methyl-3-hexen-2-one | 0.20 | 0.17 | 0.16 | 0.41 | 0.37 | 0.37 | 0.64 | 0.60 | 0.69 |
| 2-heptanone-M | 1.56 | 1.51 | 1.30 | 1.56 | 1.47 | 1.46 | 3.17 | 3.35 | 3.36 |
| cyclopentanone | 0.38 | 0.44 | 0.47 | 0.45 | 0.50 | 0.47 | 0.33 | 0.36 | 0.34 |
| acetoin | 0.16 | 0.18 | 0.18 | 0.13 | 0.18 | 0.14 | 0.06 | 0.08 | 0.07 |
| 2-butanone | 5.72 | 5.05 | 5.29 | 2.91 | 2.97 | 2.86 | 4.68 | 4.92 | 4.75 |
| 2,3-butanedione | 0.25 | 0.34 | 0.45 | 0.38 | 0.45 | 0.47 | 0.35 | 0.39 | 0.38 |
| 2-hexanone | 0.11 | 0.09 | 0.09 | 0.11 | 0.09 | 0.09 | 0.21 | 0.21 | 0.21 |
| 2,5-hexanedione | 0.68 | 0.72 | 0.76 | 0.57 | 0.72 | 0.63 | 0.67 | 0.76 | 0.73 |
| 2-pentanone-M | 0.50 | 0.55 | 0.45 | 0.08 | 0.10 | 0.09 | 0.25 | 0.31 | 0.27 |
| methyl isobutyl ketone | 0.86 | 0.78 | 0.86 | 1.09 | 1.16 | 1.13 | 1.22 | 1.31 | 1.25 |
| 2,3-pentanedione | 0.22 | 0.25 | 0.27 | 0.16 | 0.22 | 0.23 | 0.17 | 0.20 | 0.21 |
| 2-octanone | 0.32 | 0.40 | 0.36 | 0.11 | 0.14 | 0.13 | 0.10 | 0.18 | 0.11 |
| 3-heptanone | 0.06 | 0.06 | 0.05 | 0.11 | 0.09 | 0.10 | 0.41 | 0.42 | 0.46 |
| 2-pentanone-D | 2.63 | 2.73 | 2.53 | 1.92 | 1.83 | 1.76 | 3.31 | 3.43 | 3.41 |
| ***Aldehydes*** | **42.77** | **42.23** | **41.01** | **45.55** | **44.28** | **43.84** | **40.76** | **39.11** | **39.82** |
| octanal-M | 1.21 | 0.99 | 0.83 | 1.85 | 1.76 | 1.64 | 1.53 | 1.34 | 1.41 |
| octanal-D | 0.18 | 0.14 | 0.12 | 0.79 | 0.60 | 0.52 | 0.35 | 0.25 | 0.30 |
| 2-thiophenecarboxaldehyde | 0.37 | 0.67 | 0.75 | 2.11 | 2.39 | 2.71 | 0.64 | 0.38 | 0.63 |
| (E)-hept-2-enal | 0.64 | 0.33 | 0.32 | 0.66 | 0.52 | 0.55 | 0.86 | 0.62 | 0.69 |
| heptanal-D | 1.21 | 1.09 | 1.16 | 3.00 | 2.72 | 2.58 | 1.15 | 0.92 | 1.05 |
| heptanal-M | 3.06 | 2.93 | 2.98 | 2.94 | 3.02 | 2.95 | 2.50 | 2.39 | 2.44 |
| hexanal-M | 8.37 | 8.50 | 7.76 | 6.12 | 5.83 | 5.84 | 6.62 | 6.67 | 6.59 |
| hexanal-D | 7.84 | 8.73 | 8.04 | 11.47 | 10.86 | 10.96 | 10.89 | 10.04 | 10.74 |
| 3-methylbutanal | 16.92 | 16.01 | 16.23 | 13.05 | 13.23 | 12.79 | 13.40 | 13.88 | 13.22 |
| propanal | 0.14 | 0.12 | 0.11 | 0.23 | 0.21 | 0.19 | 0.17 | 0.16 | 0.15 |
| pentanal-M | 1.13 | 1.07 | 1.05 | 0.54 | 0.62 | 0.58 | 0.58 | 0.67 | 0.64 |
| 2-methyl-2-propenal | 0.33 | 0.28 | 0.24 | 0.28 | 0.27 | 0.26 | 0.24 | 0.26 | 0.24 |
| pentanal-D | 1.37 | 1.36 | 1.45 | 2.51 | 2.24 | 2.29 | 1.82 | 1.53 | 1.72 |
| ***Alcohols*** | **19.61** | **19.55** | **19.77** | **18.00** | **18.67** | **19.03** | **18.12** | **18.78** | **18.15** |
| 2-phenylethanol | 0.54 | 0.80 | 1.07 | 0.71 | 0.79 | 0.79 | 0.44 | 0.42 | 0.49 |
| linalool oxide | 1.13 | 0.96 | 1.08 | 2.34 | 2.62 | 2.90 | 1.41 | 1.27 | 1.23 |
| 2-octanol | 0.52 | 0.83 | 1.11 | 1.12 | 1.24 | 1.32 | 0.61 | 0.58 | 0.60 |
| benzyl alcohol | 0.51 | 0.56 | 0.59 | 1.01 | 1.00 | 1.04 | 1.06 | 1.15 | 1.10 |
| 1-octen-3-ol | 1.01 | 0.85 | 0.86 | 1.15 | 1.21 | 1.23 | 1.30 | 1.33 | 1.28 |
| 1-hexen-3-ol | 0.27 | 0.28 | 0.25 | 0.24 | 0.21 | 0.20 | 0.39 | 0.40 | 0.43 |
| 2,3-butanediol | 1.11 | 1.29 | 1.42 | 1.43 | 1.59 | 1.57 | 1.47 | 1.53 | 1.60 |
| 3-methylbutanol | 0.92 | 1.16 | 0.98 | 0.63 | 0.58 | 0.62 | 0.46 | 0.50 | 0.52 |
| 2-methyl-1-propanol | 1.08 | 1.29 | 1.11 | 0.49 | 0.54 | 0.54 | 0.67 | 0.75 | 0.72 |
| 2-propanol | 0.14 | 0.17 | 0.19 | 0.20 | 0.25 | 0.24 | 0.20 | 0.23 | 0.22 |
| 2-methyl-2-propanol | 10.84 | 9.85 | 9.65 | 7.61 | 7.58 | 7.37 | 8.62 | 9.02 | 8.55 |
| ethanol | 1.33 | 1.33 | 1.29 | 0.90 | 0.87 | 1.00 | 1.25 | 1.38 | 1.19 |
| n-hexanol | 0.19 | 0.17 | 0.19 | 0.17 | 0.18 | 0.21 | 0.23 | 0.23 | 0.22 |
| ***Esters*** | **4.00** | **3.96** | **4.09** | **2.98** | **3.11** | **3.13** | **3.27** | **3.39** | **3.31** |
| acetic acid ethyl ester-D | 0.19 | 0.18 | 0.18 | 0.18 | 0.18 | 0.17 | 0.16 | 0.17 | 0.16 |
| methyl propanoate | 0.51 | 0.43 | 0.46 | 0.30 | 0.33 | 0.33 | 0.48 | 0.50 | 0.46 |
| acetic acid ethyl ester-M | 0.82 | 0.77 | 0.75 | 0.54 | 0.56 | 0.54 | 0.71 | 0.74 | 0.71 |
| allyl acetate | 1.41 | 1.44 | 1.32 | 0.63 | 0.70 | 0.69 | 0.79 | 0.86 | 0.81 |
| isopropyl isobutanoate | 0.11 | 0.17 | 0.25 | 0.40 | 0.42 | 0.47 | 0.36 | 0.34 | 0.38 |
| isopropyl acetate | 0.31 | 0.40 | 0.54 | 0.39 | 0.44 | 0.46 | 0.42 | 0.45 | 0.45 |
| propyl acetate | 0.65 | 0.57 | 0.60 | 0.53 | 0.47 | 0.47 | 0.35 | 0.32 | 0.34 |
| ***S-containing compounds*** | **3.50** | **3.38** | **3.61** | **5.37** | **5.28** | **5.20** | **4.14** | **3.44** | **4.04** |
| dipropyl disulfide | 1.12 | 0.74 | 0.77 | 0.74 | 0.73 | 0.66 | 0.69 | 0.61 | 0.71 |
| furfuryl methyl sulfide | 0.31 | 0.39 | 0.34 | 2.50 | 2.34 | 2.36 | 1.14 | 0.46 | 0.96 |
| S-methyl propanethioate | 0.54 | 0.62 | 0.63 | 0.33 | 0.42 | 0.39 | 0.45 | 0.50 | 0.47 |
| allyl isothiocyanate | 0.24 | 0.37 | 0.56 | 0.46 | 0.49 | 0.52 | 0.68 | 0.72 | 0.72 |
| 1-butanethiol | 1.28 | 1.25 | 1.31 | 1.34 | 1.31 | 1.27 | 1.19 | 1.16 | 1.18 |
| ***N-containing compounds*** | **3.65** | **4.11** | **4.03** | **2.93** | **3.02** | **3.04** | **3.42** | **3.63** | **3.52** |
| pyrazine, 2-ethyl-3-methyl- | 0.56 | 0.62 | 0.63 | 1.20 | 1.14 | 1.09 | 1.69 | 1.75 | 1.71 |
| pyridine | 2.56 | 2.85 | 2.56 | 1.15 | 1.22 | 1.31 | 1.16 | 1.24 | 1.22 |
| pyrrolidine | 0.54 | 0.64 | 0.84 | 0.58 | 0.66 | 0.65 | 0.57 | 0.64 | 0.59 |
| ***Hydrocarbons*** | **1.60** | **2.14** | **2.63** | **1.79** | **2.07** | **2.19** | **1.80** | **1.86** | **1.87** |
| β-pinene | 0.24 | 0.51 | 0.70 | 0.43 | 0.46 | 0.54 | 0.29 | 0.29 | 0.28 |
| 1-Octene | 0.80 | 0.85 | 0.85 | 0.51 | 0.63 | 0.61 | 0.73 | 0.79 | 0.76 |
| styrene | 0.55 | 0.78 | 1.08 | 0.85 | 0.98 | 1.05 | 0.78 | 0.78 | 0.82 |
| ***Acids*** | **1.30** | **1.29** | **1.52** | **1.85** | **2.02** | **2.07** | **1.40** | **1.42** | **1.39** |
| 2-methylpropionic acid | 0.57 | 0.59 | 0.88 | 1.17 | 1.30 | 1.38 | 0.79 | 0.76 | 0.76 |
| acetic acid | 0.73 | 0.70 | 0.63 | 0.68 | 0.72 | 0.69 | 0.62 | 0.67 | 0.63 |
| ***Ethers*** | **2.31** | **1.95** | **2.05** | **1.56** | **1.53** | **1.46** | **2.01** | **2.01** | **1.87** |
| 1,2-dimethoxyethane | 1.62 | 1.42 | 1.44 | 1.18 | 1.18 | 1.13 | 1.37 | 1.42 | 1.37 |
| tert-butylmethylether | 0.69 | 0.53 | 0.60 | 0.38 | 0.35 | 0.33 | 0.64 | 0.59 | 0.50 |
| ***Furans*** | **0.17** | **0.21** | **0.28** | **0.37** | **0.31** | **0.34** | **0.55** | **0.48** | **0.40** |
| 2-pentylfuran | 0.17 | 0.21 | 0.28 | 0.37 | 0.31 | 0.34 | 0.55 | 0.48 | 0.40 |
| ***Others*** | **0.04** | **0.05** | **0.05** | **0.08** | **0.07** | **0.07** | **0.49** | **0.52** | **0.57** |
| hexanenitrile | 0.04 | 0.05 | 0.05 | 0.08 | 0.07 | 0.07 | 0.49 | 0.52 | 0.57 |
| ***Unidentified*** | **7.23** | **7.74** | **7.61** | **9.28** | **9.14** | **9.43** | **8.25** | **8.60** | **8.57** |
| unidentified 1 | 0.33 | 0.32 | 0.32 | 0.70 | 0.63 | 0.63 | 0.71 | 0.64 | 0.73 |
| unidentified 2 | 0.05 | 0.05 | 0.04 | 0.42 | 0.38 | 0.41 | 0.24 | 0.26 | 0.25 |
| unidentified 3 | 0.28 | 0.34 | 0.27 | 0.47 | 0.47 | 0.47 | 0.19 | 0.19 | 0.19 |
| unidentified 4 | 0.31 | 0.30 | 0.27 | 2.22 | 1.91 | 2.10 | 1.73 | 1.81 | 1.80 |
| unidentified 5 | 0.11 | 0.12 | 0.10 | 0.19 | 0.21 | 0.21 | 0.16 | 0.16 | 0.15 |
| unidentified 6 | 0.57 | 0.69 | 0.90 | 0.45 | 0.57 | 0.60 | 0.53 | 0.59 | 0.58 |
| unidentified 7 | 1.23 | 1.41 | 1.63 | 1.15 | 1.23 | 1.23 | 1.29 | 1.42 | 1.38 |
| unidentified 8 | 0.05 | 0.04 | 0.04 | 0.23 | 0.25 | 0.29 | 0.23 | 0.25 | 0.26 |
| unidentified 9 | 1.10 | 1.32 | 1.11 | 1.18 | 1.21 | 1.23 | 0.56 | 0.54 | 0.52 |
| unidentified 10 | 0.10 | 0.10 | 0.10 | 0.47 | 0.40 | 0.42 | 0.14 | 0.09 | 0.12 |
| unidentified 11 | 0.45 | 0.39 | 0.38 | 0.30 | 0.30 | 0.30 | 0.28 | 0.30 | 0.28 |
| unidentified 12 | 2.63 | 2.60 | 2.39 | 1.41 | 1.50 | 1.49 | 1.69 | 1.83 | 1.75 |

**Table S2** Detailed information on volatile compounds detected by HS-SPME-GC-MS

| **Compounds** | **Concentration (mg/kg)** | | | | | | | | |
| --- | --- | --- | --- | --- | --- | --- | --- | --- | --- |
|  | **PDZ-1** | **PDZ-2** | **PDZ-3** | **CBZ-1** | **CBZ-2** | **CBZ-3** | **DLK-1** | **DLK-2** | **DLK-3** |
| ***Aldehydes*** | **29.98** | **37.20** | **32.91** | **68.10** | **64.51** | **58.17** | **37.23** | **74.37** | **46.02** |
| Hexanal | 2.66 | 3.38 | 3.98 | 24.45 | 15.95 | 14.91 | 6.66 | 8.88 | 8.91 |
| Octanal | 1.82 | 2.53 | 2.79 | 2.27 | 4.22 | 3.00 | 1.20 | 3.94 | 2.15 |
| (E)-2-Heptenal | 0.00 | 0.00 | 0.00 | 7.21 | 6.64 | 8.32 | 3.01 | 5.88 | 5.93 |
| Nonanal | 6.52 | 9.05 | 9.44 | 6.74 | 8.22 | 5.66 | 5.62 | 11.55 | 4.52 |
| 5-Ethylcyclopent-1-necarboxaldehyde | 0.00 | 0.00 | 0.00 | 1.60 | 1.65 | 1.66 | 0.86 | 1.02 | 0.70 |
| (E,E)-2,4-Heptadienal | 0.00 | 0.00 | 0.00 | 1.43 | 0.81 | 1.49 | 0.00 | 0.00 | 0.00 |
| Decanal | 0.27 | 0.26 | 0.40 | 0.00 | 0.00 | 0.00 | 0.00 | 0.00 | 0.00 |
| (E)-2-Nonenal | 0.00 | 0.00 | 0.00 | 2.33 | 1.96 | 2.13 | 0.77 | 0.80 | 0.49 |
| Benzaldehyde | 5.98 | 7.74 | 5.60 | 5.55 | 9.06 | 7.09 | 8.08 | 15.83 | 8.26 |
| Benzeneacetaldehyde | 12.47 | 13.85 | 10.15 | 9.68 | 8.68 | 7.79 | 6.70 | 19.95 | 10.38 |
| 3-Ethylbenzaldehyde | 0.00 | 0.00 | 0.00 | 0.17 | 0.26 | 0.16 | 0.21 | 0.29 | 0.15 |
| (E)-2-Decenal | 0.26 | 0.41 | 0.55 | 1.20 | 1.56 | 1.15 | 0.91 | 1.25 | 0.99 |
| (E,E)-2,4-Nonadienal | 0.00 | 0.00 | 0.00 | 1.53 | 1.50 | 1.53 | 0.67 | 1.11 | 0.73 |
| (E)-Tetradec-2-enal | 0.00 | 0.00 | 0.00 | 0.32 | 0.39 | 0.36 | 0.00 | 0.00 | 0.00 |
| (E,Z)-2,4-Decadienal | 0.00 | 0.00 | 0.00 | 0.74 | 0.68 | 0.60 | 0.41 | 0.70 | 0.44 |
| (E,E)-2,4-Decadienal | 0.00 | 0.00 | 0.00 | 2.87 | 2.93 | 2.34 | 2.12 | 3.18 | 2.37 |
| ***Esters*** | **44.67** | **37.56** | **56.06** | **6.78** | **7.81** | **7.08** | **22.01** | **7.92** | **17.65** |
| Methyl isovalerate | 23.57 | 1.90 | 19.85 | 0.00 | 0.00 | 0.00 | 0.00 | 0.00 | 0.00 |
| Methyl hexanoate | 10.89 | 25.72 | 26.16 | 0.00 | 0.00 | 0.00 | 16.88 | 4.36 | 12.32 |
| Methyl octanoate | 5.53 | 5.07 | 4.97 | 1.84 | 2.64 | 2.62 | 2.24 | 1.15 | 2.02 |
| Ethyl octanoate | 0.29 | 0.32 | 0.30 | 1.19 | 0.93 | 0.93 | 0.00 | 0.00 | 0.00 |
| Methyl decanoate | 0.52 | 0.49 | 0.68 | 0.00 | 0.40 | 0.35 | 0.84 | 0.40 | 1.08 |
| Ethyl decanoate | 0.00 | 0.00 | 0.00 | 1.07 | 0.84 | 0.89 | 0.00 | 0.00 | 0.00 |
| Ethyl 4-ethoxy-2-oxobut-3-enoate | 0.00 | 0.00 | 0.00 | 1.63 | 1.89 | 1.35 | 0.00 | 0.00 | 0.00 |
| 1,3-Benzenediol monobenzoate | 0.24 | 0.27 | 0.25 | 0.00 | 0.00 | 0.00 | 0.00 | 0.00 | 0.00 |
| Methyl benzeneacetate | 0.89 | 1.02 | 1.06 | 0.00 | 0.00 | 0.00 | 0.00 | 0.00 | 0.00 |
| 4-Methylphenyl acetate | 1.41 | 1.49 | 1.57 | 0.00 | 0.00 | 0.00 | 1.05 | 0.91 | 1.19 |
| Ethyl 4-ethoxybenzoate | 1.33 | 1.27 | 1.21 | 1.05 | 1.11 | 0.95 | 1.00 | 1.10 | 1.05 |
| ***Acids*** | **27.70** | **20.33** | **26.70** | **28.73** | **31.97** | **24.88** | **34.80** | **31.99** | **27.41** |
| Acetic acid | 1.49 | 1.69 | 1.41 | 2.13 | 2.73 | 1.84 | 2.41 | 2.15 | 1.31 |
| 2-Methylpropanoic acid | 1.30 | 1.21 | 0.98 | 0.47 | 0.67 | 0.57 | 0.00 | 0.00 | 0.00 |
| Butanoic acid | 3.07 | 2.86 | 2.58 | 0.00 | 0.00 | 0.00 | 0.00 | 0.00 | 0.00 |
| 3-Methylbutanoic acid | 14.43 | 7.26 | 12.74 | 5.17 | 6.70 | 4.68 | 3.18 | 1.77 | 3.39 |
| Pentanoic acid | 0.00 | 0.00 | 0.00 | 0.00 | 0.00 | 0.00 | 1.18 | 1.37 | 1.01 |
| Hexanoic acid | 6.27 | 6.31 | 7.80 | 15.60 | 16.82 | 13.58 | 20.49 | 21.53 | 16.09 |
| Heptanoic acid | 0.00 | 0.00 | 0.00 | 0.00 | 0.00 | 0.00 | 1.29 | 1.22 | 0.85 |
| Octanoic acid | 1.14 | 0.99 | 1.18 | 3.92 | 3.69 | 3.26 | 4.19 | 3.29 | 3.20 |
| n-Decanoic acid | 0.00 | 0.00 | 0.00 | 1.44 | 1.37 | 0.94 | 2.06 | 0.66 | 1.56 |
| ***Alcohols*** | **6.08** | **6.82** | **9.24** | **16.93** | **22.08** | **15.80** | **16.85** | **24.87** | **15.50** |
| 1-Octen-3-ol | 3.08 | 3.87 | 5.22 | 14.67 | 19.10 | 14.00 | 13.46 | 21.09 | 13.00 |
| 4-Ethylcyclohexanol | 0.00 | 0.00 | 0.00 | 0.00 | 0.00 | 0.00 | 0.49 | 0.61 | 0.46 |
| 1-Octanol | 0.51 | 0.81 | 0.73 | 0.98 | 1.53 | 0.88 | 0.75 | 1.48 | 0.80 |
| Cyclooctyl alcohol | 0.00 | 0.00 | 0.00 | 0.32 | 0.68 | 0.32 | 0.00 | 0.00 | 0.00 |
| Benzyl alcohol | 0.20 | 0.15 | 0.26 | 0.35 | 0.37 | 0.29 | 0.96 | 0.62 | 0.61 |
| Phenylethyl alcohol | 2.30 | 1.99 | 3.02 | 0.61 | 0.40 | 0.31 | 1.19 | 1.07 | 0.62 |
| ***N-containing compounds*** | **6.97** | **8.13** | **8.01** | **10.61** | **11.83** | **9.14** | **2.85** | **2.77** | **2.45** |
| Trimethylpyrazine | 5.48 | 6.45 | 5.57 | 6.64 | 6.85 | 5.90 | 2.85 | 2.77 | 2.45 |
| 2,6-Diethylpyrazine | 0.19 | 0.21 | 0.22 | 0.00 | 0.00 | 0.00 | 0.00 | 0.00 | 0.00 |
| Tetramethylpyrazine | 1.31 | 1.48 | 2.22 | 3.97 | 4.98 | 3.24 | 0.00 | 0.00 | 0.00 |
| ***Hydrocarbons*** | **1.43** | **2.38** | **3.17** | **7.47** | **4.90** | **2.60** | **7.49** | **6.76** | **7.22** |
| Decane | 0.00 | 0.00 | 0.00 | 3.97 | 3.11 | 1.80 | 5.37 | 3.59 | 4.43 |
| 3-Methylundecane | 0.00 | 0.00 | 0.00 | 0.00 | 0.00 | 0.00 | 1.27 | 0.87 | 0.69 |
| Dodecane | 1.43 | 2.38 | 3.17 | 3.50 | 1.79 | 0.80 | 0.85 | 2.29 | 2.10 |
| ***Furans*** | **1.11** | **1.16** | **1.07** | **11.63** | **5.18** | **6.72** | **4.88** | **1.85** | **6.00** |
| 2-Pentylfuran | 0.00 | 0.00 | 0.00 | 8.26 | 3.03 | 3.20 | 4.88 | 1.85 | 6.00 |
| 2-Propylfuran | 0.00 | 0.00 | 0.00 | 3.37 | 2.15 | 3.52 | 0.00 | 0.00 | 0.00 |
| 5-Ethyldihydro-2(3H)-furanone | 1.11 | 1.16 | 1.07 | 0.00 | 0.00 | 0.00 | 0.00 | 0.00 | 0.00 |
| ***Ketones*** | **0.00** | **0.00** | **0.00** | **3.32** | **3.08** | **2.75** | **1.98** | **2.92** | **2.74** |
| 1-Hepten-3-one | 0.00 | 0.00 | 0.00 | 0.00 | 0.00 | 0.00 | 1.37 | 2.26 | 2.22 |
| 3,5-Octadien-2-one | 0.00 | 0.00 | 0.00 | 3.32 | 3.08 | 2.75 | 0.60 | 0.67 | 0.52 |
| ***S-containing compounds*** | **3.07** | **3.68** | **2.52** | **0.00** | **0.00** | **0.00** | **0.00** | **0.00** | **0.00** |
| Dimethyl trisulfide | 2.10 | 2.39 | 1.40 | 0.00 | 0.00 | 0.00 | 0.00 | 0.00 | 0.00 |
| Methional | 0.96 | 1.29 | 1.13 | 0.00 | 0.00 | 0.00 | 0.00 | 0.00 | 0.00 |
| ***Phenols*** | **0.00** | **0.00** | **0.00** | **0.00** | **0.00** | **0.00** | **2.06** | **1.57** | **0.83** |
| 4-(1-Methylpropyl)phenol | 0.00 | 0.00 | 0.00 | 0.00 | 0.00 | 0.00 | 0.24 | 0.40 | 0.31 |
| p-Cresol | 0.00 | 0.00 | 0.00 | 0.00 | 0.00 | 0.00 | 1.82 | 1.17 | 0.53 |
| ***Others*** | **0.00** | **0.00** | **0.00** | **0.00** | **0.00** | **0.00** | **0.98** | **4.96** | **2.08** |
| 2-Methylpentanoic anhydride | 0.00 | 0.00 | 0.00 | 0.00 | 0.00 | 0.00 | 0.98 | 4.96 | 2.08 |

**Table S3** The ROAV results based on differential compounds identified by HS-GC-IMS

| **ID** | **Compounds^a^** | **CAS** | **Relative content (%)** | | | **Odor thresholds**  **(mg/kg)^b^** | **Odor characteristics** | **ROAV** | | | |
| --- | --- | --- | --- | --- | --- | --- | --- | --- | --- | --- | --- |
|  |  |  | **PDZ** | **CBZ** | **DLK** |  |  | **PDZ** | **CBZ** | **DLK** | **Average** |
| 1 | 1-Octen-3-ol | 3391-86-4 | 0.90 | 1.20 | 1.30 | 0.001 | mushroom, earthy | 69.57 | 91.99 | 100.00 | 87.19 |
| 2 | Hexanal | 66-25-1 | 16.40 | 22.19 | 21.11 | 0.075 | green, fatty | 16.81 | 22.75 | 21.64 | 20.40 |
| 3 | Hexanenitrile | 628-73-9 | 0.04 | 0.07 | 0.53 | 0.0032^c^ | pungent, fatty | 1.03 | 1.77 | 12.62 | 5.14 |
| 4 | Octanal | 124-13-0 | 2.02 | 3.50 | 2.86 | 0.32 | fatty, citrus | 0.49 | 0.84 | 0.69 | 0.67 |
| 5 | 3-Methyl-1-butanol | 123-51-3 | 1.02 | 0.61 | 0.49 | 0.1 | alcoholic, fermented | 0.45 | 0.75 | 0.54 | 0.58 |
| 6 | Pentanal | 110-62-3 | 1.39 | 2.35 | 1.69 | 0.24 | green, fatty | 0.78 | 0.47 | 0.38 | 0.54 |
| 7 | 2-Pentanone | 107-87-9 | 5.25 | 3.67 | 6.77 | 1.38^c^ | fruity, sweet | 0.29 | 0.20 | 0.38 | 0.29 |
| 8 | 4-Methyl-2-pentanone | 108-10-1 | 0.83 | 1.12 | 1.26 | 0.3 | solvent, sweet | 0.21 | 0.29 | 0.32 | 0.28 |
| 9 | Heptanal | 111-71-7 | 1.16 | 2.76 | 1.04 | 0.5 | fatty, waxy | 0.18 | 0.43 | 0.16 | 0.25 |
| 10 | 3-Heptanone | 106-35-4 | 0.06 | 0.10 | 0.43 | 0.08^c^ | fruity, creamy | 0.06 | 0.10 | 0.41 | 0.19 |
| 11 | 2-Ethyl-3-methylpyrazine | 15707-23-0 | 0.60 | 1.14 | 1.72 | 0.5^c^ | roasted, nutty | 0.09 | 0.18 | 0.26 | 0.18 |
| 12 | 2-Heptanone | 110-43-0 | 0.19 | 0.31 | 0.75 | 0.3 | fruity, creamy | 0.05 | 0.08 | 0.19 | 0.11 |
| 13 | tert-Butanol | 75-65-0 | 10.12 | 7.52 | 8.73 | 8.2 | alcoholic, solvent | 0.09 | 0.07 | 0.08 | 0.08 |
| 14 | 2-Methyl-1-propanol | 78-83-1 | 1.16 | 0.53 | 0.71 | 1 | alcoholic, fermented | 0.09 | 0.04 | 0.05 | 0.06 |
| 15 | 1-Hexen-3-ol | 4798-44-1 | 0.27 | 0.22 | 0.41 | 0.5 | green, mushroom | 0.04 | 0.03 | 0.06 | 0.05 |
| 16 | Benzyl alcohol | 100-51-6 | 0.55 | 1.02 | 1.10 | 2.54^c^ | floral, sweet | 0.02 | 0.03 | 0.03 | 0.03 |
| 17 | 2-Butanone | 78-93-3 | 5.35 | 2.91 | 4.79 | 40 | sweet, solvent | 0.01 | 0.01 | 0.01 | 0.01 |
| 18 | Furfuryl methyl sulfide | 1438-91-7 | 0.35 | 2.40 | 0.85 | —^d^ |  | — | — | — | — |
| 19 | Linalool oxide | 1365-19-1 | 1.05 | 2.62 | 1.30 | —^d^ |  | — | — | — | — |
| 20 | Allyl acetate | 591-87-7 | 1.39 | 0.67 | 0.82 | —^d^ |  | — | — | — | — |
| 21 | 5-Methyl-3-hexen-2-one | 5166-53-0 | 0.18 | 0.38 | 0.65 | —^d^ |  | — | — | — | — |

^a^ Monomers and dimers of the same compound were combined to calculate its ROAV.

^b^ Odor thresholds for all compounds were obtained from Compilations of Odour Threshold Values in Air, Water and Other Media (L.J. van Gemert, 2011).

^c^ The odor thresholds of these compounds were obtained from water, while the remaining compounds were obtained from oil.

^d^ “—” indicates that the olfactory threshold of this compound has not been determined.

**Table S4** The OAV results based on differential compounds identified by HS-SPME-GC-MS

| **ID** | **Compound name** | **CAS** | **Concentration (mg/kg)** | | | **Odor thresholds**  **(mg/kg)^a^** | **Odor characteristics** | **OAV** | | | |
| --- | --- | --- | --- | --- | --- | --- | --- | --- | --- | --- | --- |
|  |  |  | **PDZ** | **CBZ** | **DLK** |  |  | **PDZ** | **CBZ** | **DLK** | **Average** |
| 1 | 1-Octen-3-ol | 3391-86-4 | 4.06 | 15.92 | 15.85 | 0.001 | mushroom, earthy | 4055.73 | 15922.92 | 15850.34 | 11943.00 |
| 2 | Methyl 3-methylbutyrate | 556-24-1 | 18.44 | 0.00 | 0.00 | 0.0044^b^ | fruity, tropical | 4190.82 | ND | ND | 4190.82 |
| 3 | Phenylacetaldehyde | 122-78-1 | 12.16 | 8.72 | 12.34 | 0.025 | floral, honey | 486.23 | 348.66 | 493.65 | 442.84 |
| 4 | 3-Methylbutanoic acid | 503-74-2 | 11.48 | 5.52 | 2.78 | 0.041 | sour, cheesy | 279.98 | 134.53 | 67.84 | 160.78 |
| 5 | Methyl hexanoate | 106-70-7 | 17.59 | 0.00 | 11.18 | 0.07^b^ | fruity, pineapple | 251.30 | ND | 159.78 | 205.54 |
| 6 | Benzaldehyde | 100-52-7 | 6.44 | 7.23 | 10.72 | 0.06 | nutty, almond | 107.33 | 120.52 | 178.72 | 135.52 |
| 7 | Hexanal | 66-25-1 | 3.34 | 18.44 | 8.15 | 0.075 | green, fatty | 44.54 | 245.83 | 108.66 | 133.01 |
| 8 | 1-Hepten-3-one | 2918-13-0 | 0.00 | 0.00 | 1.95 | 0.007 | mushroom, green | ND | ND | 278.90 | 278.90 |
| 9 | (E)-2-Heptenal | 18829-55-5 | 0.00 | 7.39 | 4.94 | 0.05 | green, fatty | ND | 147.77 | 98.80 | 123.29 |
| 10 | 2-Pentylfuran | 3777-69-3 | 0.00 | 4.83 | 4.24 | 0.1 | beany, nutty | ND | 48.29 | 42.44 | 45.36 |
| 11 | Hexanoic acid | 142-62-1 | 6.79 | 15.33 | 19.37 | 0.7 | sour, cheesy | 9.70 | 21.91 | 27.67 | 19.76 |
| 12 | Trimethylpyrazine | 14667-55-1 | 5.83 | 6.46 | 2.69 | 0.29 | roasted, nutty | 20.11 | 22.29 | 9.27 | 17.22 |
| 13 | 2-Propylfuran | 4229-91-8 | 0.00 | 3.01 | 0.00 | 6 | roasted, nutty | 0.00 | 0.50 | 0.00 | 0.17 |
| 14 | Tetramethylpyrazine | 1124-11-4 | 1.67 | 4.06 | 0.00 | 38 | roasted, nutty | 0.04 | 0.11 | 0.00 | 0.05 |
| 15 | Decane | 124-18-5 | 0.00 | 2.96 | 4.47 | 4000 | waxy, fatty | 0.00 | 0.00 | 0.00 | 0.00 |
| 16 | 2-Methylvaleric anhydride | 63169-61-9 | 0.00 | 0.00 | 2.67 | —^c^ | sour, pungent | — | — | — | — |
| 17 | 3,5-Octadien-2-one | 38284-27-4 | 0.00 | 3.05 | 0.59 | —^c^ | mushroom, fatty | — | — | — | — |

^a^ Odor thresholds for all compounds were obtained from Compilations of Odour Threshold Values in Air, Water and Other Media (L.J. van Gemert, 2011).

^b^ The odor thresholds of these compounds were obtained from water, while the remaining compounds were obtained from oil.

^c^ “—” indicates that the olfactory threshold of this compound has not been determined.
